# Effectiveness of Neurofeedback-Assisted and Conventional 6-Week Web-Based Mindfulness Interventions on Mental Health of Chinese Nursing Students: Randomized Controlled Trial

**DOI:** 10.2196/71741

**Published:** 2025-05-23

**Authors:** Shu Jing, Zhenwei Dai, Xiaoyang Liu, Xuelin Yang, Jinglei Cheng, Tianming Chen, Zihang Feng, Xin Liu, Fenghe Dong, You Xin, Zhuoyan Han, Haiyan Hu, Xiaoyou Su, Chen Wang

**Affiliations:** 1 School of Population Medicine and Public Health Chinese Academy of Medical Sciences & Peking Union Medical College Beijing China; 2 Peking University Sixth Hospital Peking University Beijing China; 3 Peking University Institute of Mental Health Peking University Beijing China; 4 National Health Commission Key Laboratory of Mental Health Peking University Beijing China; 5 National Clinical Research Center for Mental Disorders Peking University Sixth Hospital Beijing China; 6 School of Nursing and Institute of Nursing Research, School of Medicine Zhejiang University Hangzhou China; 7 School of Nursing, Chinese Academy of Medical Sciences & Peking Union Medical College Beijing China; 8 College of Computer Science and Technology, Zhejiang University Hangzhou China; 9 The State Key Laboratory of Brain-machine Intelligence, Zhejiang University Hangzhou China; 10 School of Translation and Interpreting, Beijing Language and Culture University Beijing China; 11 Shenzhen Maternity and Child Healthcare Hospital, Southern Medical University Shenzhen China; 12 State Key Laboratory of Respiratory Health and Multimorbidity Beijing China

**Keywords:** online mindfulness, neurofeedback, nursing student mental health, randomized controlled trial, depression, anxiety

## Abstract

**Background:**

Nursing students experience disproportionately high rates of mental health challenges, underscoring the urgent need for innovative, scalable interventions. Web-based mindfulness programs, and more recently, neurofeedback-enhanced approaches, present potentially promising avenues for addressing this critical issue.

**Objective:**

This study aimed to explore the effectiveness of the neurofeedback-assisted online mindfulness intervention (NAOM) and the conventional online mindfulness intervention (COM) in reducing mental health symptoms among Chinese nursing students.

**Methods:**

A 3-armed randomized controlled trial was conducted among 147 nursing students in Beijing, China, using a 6-week web-based mindfulness program. Participants received NAOM, COM, or general mental health education across 6 weeks. Electroencephalogram and validated tools such as the Patient Health Questionnaire and the Generalized Anxiety Disorder Questionnaire were used to primarily assess symptoms of depression and anxiety at baseline, immediately after the intervention, and at 1 and 3 months after the intervention. Generalized estimating equations were used to evaluate the effects of intervention and time.

**Results:**

A total of 155 participants enrolled in the study, and 147 finished all assessments. Significant reductions in the symptoms of depression, anxiety, and fatigue were observed in the NAOM (mean difference [MD]=−3.330, Cohen *d*=0.926, *P*<.001; MD=−3.468, Cohen *d*=1.091, *P*<.001; MD=−2.620, Cohen *d*=0.743, *P*<.001, respectively) and the COM (MD=−1.875, Cohen *d*=0.490, *P*=.03; MD=−1.750, Cohen *d*=0.486, *P*=.02; MD=−2.229, Cohen *d*=0.629, *P*=.01, respectively) groups compared with the control group at postintervention assessment. Moreover, the NAOM group showed significantly better effects than the COM group in alleviating depressive symptoms (MD=−1.455; Cohen *d*=0.492; *P*=.04) and anxiety symptoms (MD=−1.718; Cohen *d*=0.670; *P*=.04) and improving the level of mindfulness (MD=−3.765; Cohen *d*=1.245; *P*<.001) at the postintervention assessment. However, no significant difference except for the anxiety symptoms was observed across the 3 groups at the 1- and 3-month follow-ups.

**Conclusions:**

This 6-week web-based mindfulness intervention, both conventional and neurofeedback-assisted, effectively alleviated mental health problems in the short term among nursing students. The addition of neurofeedback demonstrated greater short-term benefits; however, but these effects were not sustained over the long term. Future research should focus on long-term interventions using a more robust methodological approach.

**Trial Registration:**

Chinese Clinical Trial Registry (ChiCTR) ChiCTR2400080314; https://www.chictr.org.cn/bin/project/edit?pid=211845

## Introduction

### Background

Mental health is a vital dimension of human well-being, and the global importance of mental well-being is now well recognized, particularly within the health care professions [1]. Research suggested that medical students are at higher risk of experiencing numerous mental health problems compared with students in other academic disciplines [2]. Due to the overwhelming academic pressure, mandatory clinical practicum and licensing exams, immature or emotion-centered stress coping strategies, confronting patients’ painful experiences and negative emotions, and the global infectious disease epidemic in recent years, medical students, especially those majoring in nursing, face severe mental health challenges and experience higher rates of psychological disorders compared to peers of a similar age [2-4]. While previous meta-analyses have documented high rates of mental distress among medical students in general during the COVID-19 pandemic (38%-41%) [5,6], specific reviews examining the efficacy of web-based mindfulness intervention for nursing students with neurofeedback support are scarce, indicating an important gap in current research. Notably, nursing students showed higher rates of mental health problems compared to medical students in other fields, as they are faced with more serious mental health challenges due to social prejudice from their families and peers regarding their future profession [7]. According to a meta-analysis of 13,247 nursing students, the prevalence of depressive symptoms among them was 52%, far higher than that of the entire medical student population and the global historical data [8]. In China, a cross-sectional study revealed that the prevalence of depressive and anxiety symptoms among Chinese nursing students in 2020 was 56.4% and 55%, highlighting the severity of this issue [9].

Mental health problems could lead to insomnia; interpersonal difficulties; impaired memory; and reduced concentration, self-efficacy, and learning efficiency, which seriously affect both physical and mental health, academic proficiency, and professional development of nursing students [10-13]. In severe cases, mental health problems may lead to nonsuicidal self-injury, suicidal ideation, and even suicide among nursing students [14]. In addition, studies have also found a positive association between mental health and professional identity among nursing students [15,16], and mental health problems could increase the dropout intention of nursing students and reduce their willingness to engage in nursing as their occupation after graduation, further contributing to the long-term shortage of nursing professionals [17]. Following the described phenomenon, nursing students should be preventively or incipiently assisted through effective interventions in maintaining their mental health and preventing the development of mental disorders.

Mindfulness intervention is an emerging strategy that has a positive effect on reducing psychological distress and improving the mental health of nursing students. Mindfulness is defined as the intentional and conscious awareness of the present moment, without judgment or reaction. [18,19]. It helps individuals become aware of both internal sensations, thoughts and feelings, as well as their external surroundings, allowing them to keep calm, reduce stress, and respond with conscious choice rather than automatic reaction [20]. Mindfulness interventions, including meditation, yoga, and other forms, have been universally acknowledged as effective protective approaches for promoting mental health across various populations [21-23]. Studies conducted among adolescents have demonstrated that mindfulness plays a critical role in emotional regulation and exerts a positive influence on improving mental health in this population [24]. Mindfulness interventions have been shown to effectively reduce stress, depression, and anxiety symptoms in nursing students [25,26]. A meta-analysis conducted with 1579 studies showed that mindfulness interventions comprised the largest sample sizes and displayed the highest level of evidence compared to other interventions that aimed at alleviating the mental health problems of nursing students [27]. In addition, nursing students favored web-based mental health interventions over face-to-face mental consultations for mental health support owing to fears of stigmatization, shame, barriers of time and place, and distrust of intervention offers [28,29]. Web-based mindfulness interventions could overcome some of the aforementioned barriers as they can be accessed anonymously from anywhere and at any time and can be used individually in a safe setting [29,30], which is an ideal and promising approach to promote mental health and alleviate the mental problems of nursing students. In addition, as evidenced by previous studies, the long-term effects of web-based mindfulness interventions are still unclear. Some studies have shown the efficacy of mindfulness interventions in alleviating stress, depression, and anxiety among medical students, with sustained effects persisting for 6 months after the intervention [31], but some studies have not found similar results [32]. Therefore, our study conducted a 3-month follow-up to explore the long-term effects of the web-based mindfulness interventions.

Neurofeedback, based on the principle of operant conditioning of brain activity, provides real-time feedback on individuals’ brain activity and is theorized to enhance the effects of mindfulness practice by reinforcing neural patterns associated with focused attention and relaxation [33]. In recent years, neurofeedback has been applied to the treatment of neurodevelopmental disorders and has demonstrated good effects and feasibility. For instance, alpha or theta neurofeedback rehabilitation has been proven to effectively improve the attention and working memory in female students with learning disabilities [34,35]. So far, neurofeedback has also been used to assist mindfulness interventions, and neuroscience research has identified neurophysiological changes in electroencephalogram (EEG) during mindfulness interventions, such as the increased power in alpha and gamma waves [36,37]. Therefore, changes in EEG can reflect the practitioner’s state during mindfulness meditation (focused attention or wandering thoughts), and neurofeedback-assisted mindfulness meditation has been developed based on this [38]. Basic science studies have explored that brain potentials following neurofeedback could reflect the encoding of reinforced patterns in the brain, which augment and optimize the effects of mindfulness mediation [39]. A neurofeedback experiment has found that the neurofeedback mindfulness regulation can affect the brain activity pattern of patients with anxiety disorder and alleviate anxiety symptoms [36]. However, the evidence on the effectiveness of the neurofeedback-assisted mindfulness on nursing students’ mental health promotion remains limited, and few studies have focused on this population. In addition, existing studies on the neurofeedback-assisted mindfulness intervention mainly evaluated its short-term effects (immediately after the intervention) and lacked longer-term follow-ups, which limited the robustness of the evidence of effectiveness and restricted the wider use of neurofeedback-assisted mindfulness.

Identifying effective intervention methods for mental health problems in nursing students will provide valuable insights for health educators, managers, and policy makers on how to prevent the occurrence of mental health problems in nursing students through daily interventions, as well as provide timely and effective measures for at-risk students.

### Objectives

On the basis of this, our study aimed to examine the short- and long-term effectiveness of the neurofeedback-assisted online mindfulness intervention (NAOM) and conventional online mindfulness intervention (COM) in reducing mental health problems among nursing students in China. We hypothesized that (1) both COM and NAOM will have a greater reduction in depression and anxiety than the control group and (2) NAOM will have a greater reduction in anxiety and depression and an increase in mindfulness than COM.

## Methods

### Trial Design

A 3-armed, parallel-group randomized controlled trial was conducted to compare the NAOM, COM, and control groups, while acknowledging the impossibility of full blinding of participants due to the nature of interventions. A total of 172 nursing students completed the recruitment questionnaire (the baseline survey), of which 155 voluntarily participated in the subsequent intervention, with a 1:1:1 distribution across 3 groups: the NAOM group, the COM group, and the control group. In this study, we also collected resting state EEG before and after the intervention from all participants, and relevant results will be reported in other articles. The trial was prospectively registered on the Chinese Clinical Trial Registry (ChiCTR2400080314). This study was conducted in accordance with the preregistered protocol, with no deviations from the planned design, interventions, or outcome assessments.

### Development of a 6-Week Web-Based Mindfulness Intervention Program

Experienced epidemiologists and psychologists jointly discussed and designed a 6-week web-based mindfulness intervention program for this study. The program consisted of 6 modules, each consisting of two 40-minute sessions per week, which included 12 instructional videos and 9 audios, covering 4 key domains of mindfulness: attention control, self-awareness, acceptance, and emotion regulation. The contents of audio and video included theoretical knowledge, different types of meditation practice, mindful eating, mindful stretching, and mindful yoga, which were recorded by a psychiatrist certified in the Mindfulness Facilitation Training program at the Mindful Awareness Research Center at University California, Los Angeles (UCLA), specifically for mindfulness practice. This program obtained a work registration certificate from the National Copyright Administration of the People’s Republic of China (registration GZDZ-2021-I-00193781) and was delivered via a mobile app in this study. Table 1 provides a description of this course.

**Table 1:** The content of the 6-week web-based mindfulness intervention used in this study.

Week	Theme of each session	Theoretical content	Mindfulness practice
1	Mastering stress, the first sip of mindfulness	Introduce the impact of stress on health and how to effectively manage stress. Explain the 5 facets of mindfulness and the benefits of mindfulness practice Introduce the application of mindfulness practice in daily life and previous findings of mindfulness-based interventions	Mindful eating practice Mindful breathing meditation
2	Savoring mindfulness, embracing the now	Introduce the definition and practice methods of mindfulness Explain the importance and methods of mindfulness practice; introduce participants to talk to their bodies, relieve their pressure, and relax themselves	Mindful body scan practice 3-min breathing space practice
3	Tuning into body and mind, reaping restful sleep	Introduce the impact and factors of insomnia as well as the therapeutic effect of mindfulness therapy on insomnia Briefly describe the application of mindfulness practice in regulating sleep patterns and the brain’s mode of thoughts	Mountain meditation Mindful sleep meditation
4	Riding thoughts and feelings, like tides ebbing and flowing	Introduce and recognize negative emotions, transform inner states, and regulate emotions through mindfulness, focusing on mental activities Teach the importance of emotional management and the role of mindfulness meditation in it; introduce mindful living with thoughts, using the “STOP” and “RAIN” principles to deal with a storm of thoughts and emotions	Mindful walking meditation Lake meditation
5	Moving with mindfulness, loosening grip on stress	Introduce the importance of mindfulness practice in dealing with negative emotions and the methods of practicing yoga; explain the identification of avoidance response, allowing, and letting it go Describe the impact of emotions on our bodies and introduce mindful movement for relaxing the body and mind	Mindful yoga practice
6	Mindfulness as a raft, navigating joyful living	Introduce the concept of the “tired funnel” and explore how to balance daily life by practicing mindful living, especially during challenging times; discuss strategies for staying present and living a mindful, intentional life. Summarize the application of mindfulness practice in dealing with difficulties and negative emotions in daily life	Sounding meditation Loving and kindness meditation

### Sample Size

On the basis of previous research regarding the impact of web-based mindfulness interventions on enhancing mindfulness levels among Chinese nursing students [32], the sample size was calculated via a multigroup mean difference (MD) comparison approach. Specifically, the sample size was calculated via the Hsu (with best) multiple comparison test by the Power Analysis and Sample Size 14, with α=.05, power=0.80, the MDs between different groups=6.139, and the common SD within a group was assumed to be 7.16, resulting in a total sample size of 138 (46 in each group). Accounting for a 10% dropout rate, the final sample size was planned to be 153 participants.

### Participant Recruitment

Participants were recruited from a faculty of nursing at a medical college in Beijing, China, using WeChat (Tencent Holdings Ltd) and through offline publicity from March 1 to March 16, 2024. Researchers distributed digital posters through WeChat groups and put up printed posters in student dormitories, lecture halls, and classrooms to provide information regarding this study to nursing students. Interested students could scan the QR code on the posters to fill out a recruitment questionnaire (baseline survey), which collected the demographic data, baseline mental health status, and informed consent from the potential participants of the subsequent intervention. The entire recruitment process was automated to eliminate human contact and maintain scalability. The eligibility criteria were as follows: (1) full-time nursing students aged >18 years; (2) ability to independently cooperate to complete various questionnaire assessments and EEG collection; (3) possession of a mobile phone with functional Bluetooth and connection to the internet at any time; (4) right-handedness (considering that the brain structures and EEG patterns between left- and right-handed individuals are different) [40]; (5) no mindfulness-related intervention or training received within 6 months before the enrollment in this study; and (6) willingness to participate throughout the entire process, providing informed consent, and signing the electronic version of the informed consent form as required by the ethics committee. Those who were in other health promotions or clinical intervention studies, suspended or dropped out of school during the research period, or had obvious scars on the forehead or other conditions unsuitable for EEG collection were excluded.

### Procedure

**Figure 1:**
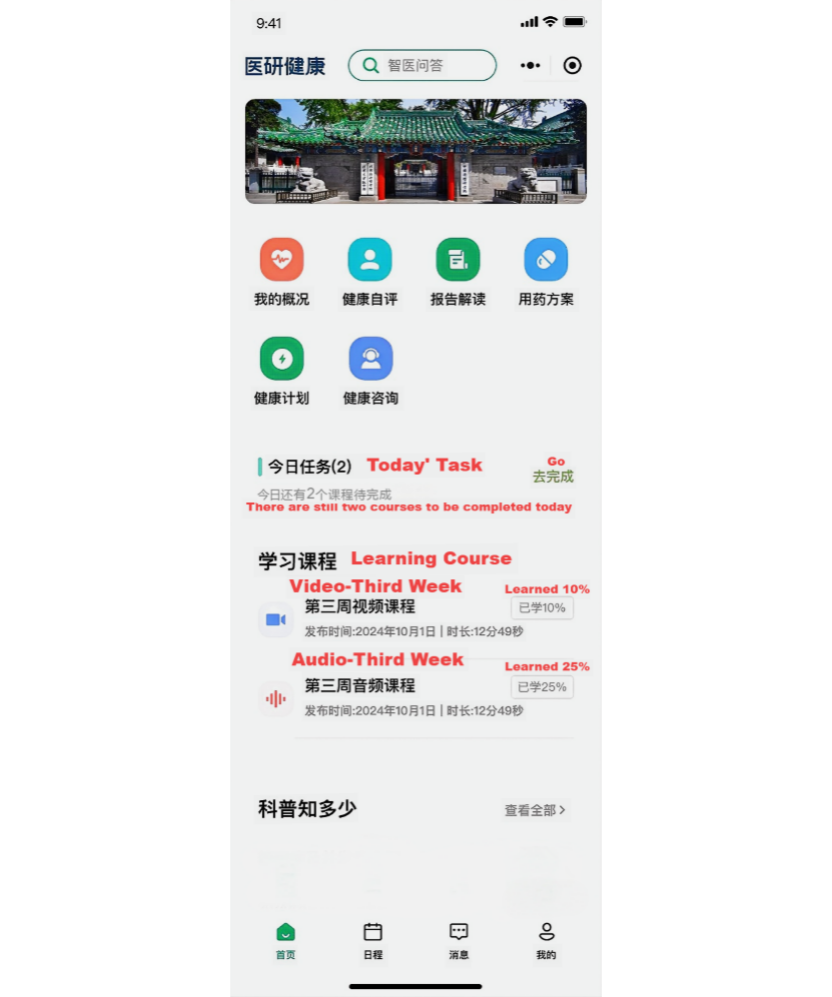
Interface of the Peking Union Medical College Questionnaire Survey applet.

Once a potential participant met the inclusion and exclusion criteria, provided informed consent, and finished the baseline questionnaire and EEG acquisition, the individual was recruited and scheduled in the study. Researchers then enter the participant’s personal information into the “Peking Union Medical College Questionnaire Survey” (PUMCQS) applet and conduct randomization. Eligible participants were then assigned to 1 of 3 groups and given access to the intervention materials within 1 week after the assignment. Participants in the NAOM group and the COM group received the 6-week web-based mindfulness intervention, and those in the control group were provided with general mental health education materials through the PUMCQS applet during the same period. This applet console could track the viewing and listening time of each participant, and reminder messages were sent to participants who had not completed the learning materials 3 days before the deadline. Assessments were conducted at 3 time points: immediately after the intervention, then again at 1 month and 3 months later. All participants received automatic messages with a follow-up questionnaire link, and those who did not complete the questionnaire within 3 days would also continuously receive reminder messages sent by the PUMCQS applet. The interface of the PUMCQS applet is shown in Figure 1. Other supporting functions of the applet, such as health self-assessment and health science popularization, were not accessible until the end of the study.

### Intervention

#### Intervention of the COM

Participants assigned to the COM group were required to participate in the aforementioned 6-week web-based mindfulness program, where they needed to take and practice 2 lessons each week, with each lesson lasting approximately 30 to 40 minutes.

#### Intervention of the NAOM

**Figure 2:**
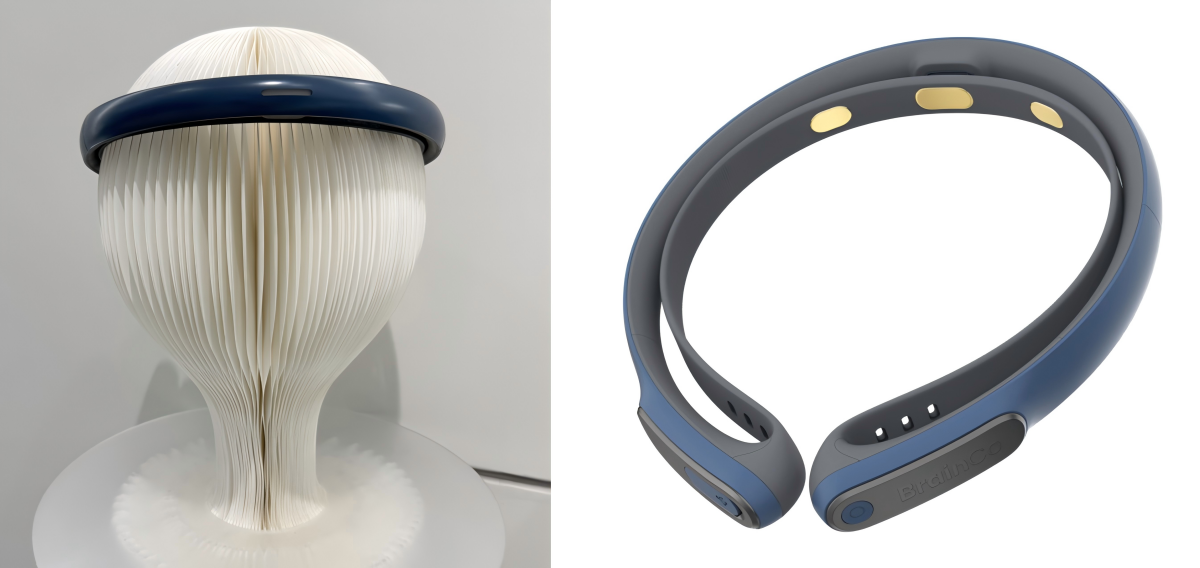
The “Focuszen” headband.

**Figure 3:**
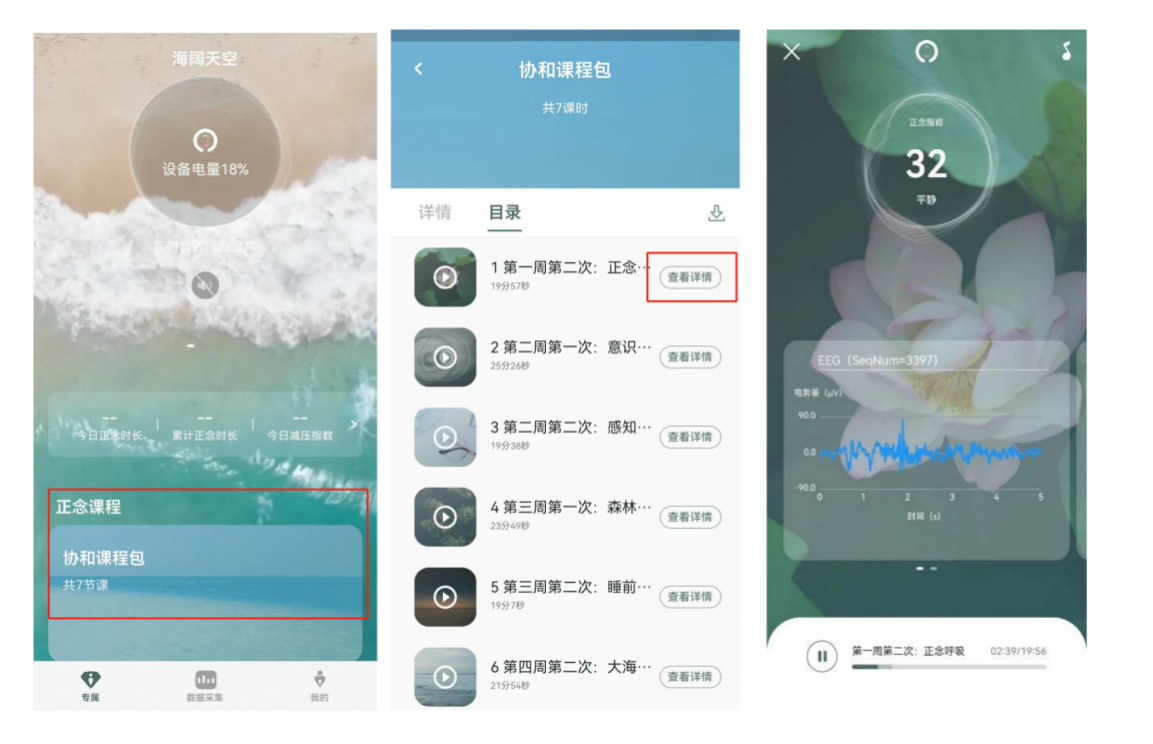
Images from the “OxyZen” mobile app used in the neurofeedback-assisted web-based mindfulness intervention.

Except for participating in the same 6-week web-based mindfulness program, participants in the NAOM group used the FocusZen headband (Zhejiang Qiangnao Technology Co, Ltd) for 6 mediation audios, which included 3 electrodes placed at prefrontal cortex locations (Fp1, Fp2, and Fpz) and generated a mindfulness index ranging from 0 to 100 based on a convolutional neural network and proprietary artificial intelligence models. The FocusZen headband and its companion app named “OxyZen” were developed by Zhejiang Qiangnao Technology Co, Ltd, which could continuously collect and process the EEG signals of the participants at a sampling rate of 250 Hz. The headband and the mobile app used in this study are shown in Figures 2 and 3, respectively.

**Figure 4:**
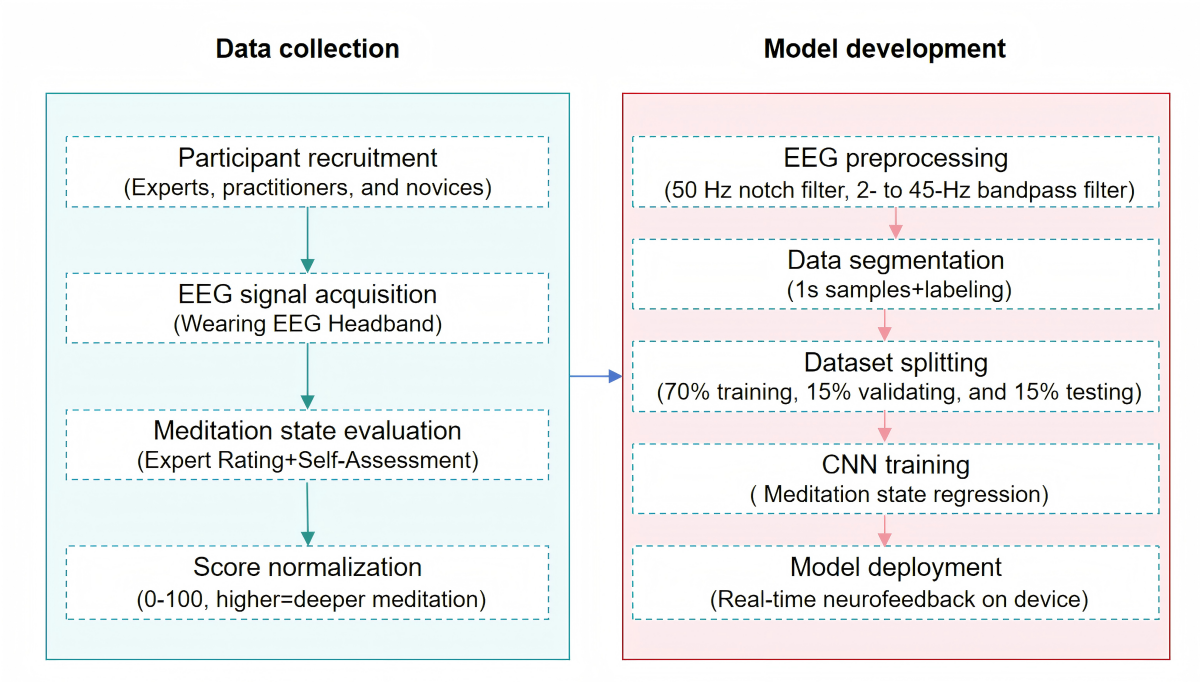
The development of the meditation neurofeedback algorithm. CNN: convolutional neural network; EEG: electroencephalogram.

Ocular, muscular, and other types of artifacts can be detected by an automatic removal algorithm in the FocusZen headband. The digitized EEG data are processed on the host device to generate a mindfulness index ranging from 0 to 100, calculated using a proprietary meditation neurofeedback algorithm embedded in the “OxyZen” app. The development of the algorithm consisted of 3 core phases: dataset construction, preprocessing, and model training, which are shown in Figure 4. For dataset construction, meditation experts, experienced practitioners, and novices with no previous meditation experience were recruited. Each participant wore the EEG headband while meditating, during which their EEG signals were recorded. After the meditation session, participants provided a subjective rating of their meditation state, while experimenters independently assessed their meditation state and adjusted the subjective scores accordingly. To ensure consistency and comparability, all ratings were normalized to a scale of 0 to 100, where higher scores indicated a deeper meditation state. This dataset provided high-quality labeled samples for subsequent model training. In the preprocessing stage, the collected EEG signals underwent filtering and denoising, including a 50-Hz notch filter to remove power line interference and a 2- to 45-Hz band-pass filter to eliminate artifacts and irrelevant signals. A sliding window approach was then applied to segment the signals into 1-second time slices. Each segment was assigned a corresponding meditation state label based on its evaluation score. After preprocessing, these EEG data were used for model training. The partitioning of the dataset followed a 7:1.5:1.5 ratio; data from 70% of the participants were used in the training set, data from 15% of the participants were used in the validation set, and data from 15% of the participants were used in the test set. A convolutional neural network was used for regression, with model performance enhanced through hyperparameter optimization and architectural refinements. After optimization, the final model was trained on the complete dataset to improve generalizability. Ultimately, the trained model was deployed on the FocusZen headband and the “OxyZen” app, enabling real-time EEG decoding and meditation state feedback.

A total of 6 mindfulness meditation audios were conducted using the “OxyZen” app, during which the participants were required to wear the FocusZen headband and collect their EEG data during meditation exercises. Upon completing each meditation practice audio, participants obtained a neurofeedback report with curve plots that visually display their mindfulness index and multidimensional brain activity analyses, providing an objective assessment of their mindfulness practice status and actionable insights to help optimize their practice for future sessions. All participants in this group received a training on the use of the FocusZen headband and the “OxyZen” app before the intervention. For the remaining videos and audios that did not involve meditation practice, participants in the NAOM group only needed to complete the web-based program through the PUMCQS applet, similar to the COM group.

#### Intervention of the Control Group

Following the baseline assessment, participants in the control group received 6 weeks of general mental health education, delivered in 2 lessons each week, similar to the other groups. Materials used in the control group were also audios and videos, covering theoretical knowledge about mental health, and common suggestions for dealing with mental problems, such as talking to family and friends, developing a hobby, and diverting attention. No mindfulness contents were included in the materials. In addition, participants in this group were given access to the full 6-week web-based mindfulness program after completing the 3-month follow-up survey.

### Randomization and Blinding

Randomization and allocation were automated and carried out independently. All participants were randomized (1:1:1 allocation ratio) using a blocked randomization sequence via the PUMCQS applet, and the trial coordinators of this study enrolled the participants after the randomization. Participants and the trial coordinators were not blinded to the group allocation due to the intrinsic limitations in intervention methods. All other members of the research team, including data analysts, were blinded to the group allocation during the intervention and the follow-ups. The groups were labeled as “A,” “B,” and “C” during the statistical analysis.

### Ethical Considerations

This study was approved by the Ethics Committee of Chinese Academy of Medical Sciences (CAMS&PUMC-IEC-2024-002) in January 2024. Electronic informed consent was obtained from all participants, with explicit emphasis on voluntary participation and the right to withdraw at any time and without giving any reason. Data were deidentified by replacing direct identifiers with unique codes and stored securely on encrypted systems. Participants were compensated with permanent free access to the mindfulness course offered in this study, a compensation method approved by the ethics committee.

### Outcomes

#### Primary Outcomes

##### Depressive Symptoms

Depressive symptoms were assessed by the 9-item Patient Health Questionnaire (PHQ-9), which was developed by Kroenke et al [41] in 2001 and has been validated among various Chinese populations [42,43]. The items of the PHQ-9 are scored on a 4-point Likert scale with a range of 0 to 3. Higher total scores indicated higher levels of depressive symptoms. Cronbach α of the instrument was 0.881, 0.860, 0.906, and 0.912, respectively, for the 4 assessments.

##### Anxiety Symptoms

The Generalized Anxiety Disorder Questionnaire (GAD-7), comprising 7 items and measured on a 4-point Likert scale from 0 to 3, was used to assess participants’ anxiety symptoms over the past 2 weeks. The scores of the instrument range from 0 to 21, and higher total scores indicated higher levels of anxiety [44]. This instrument has been demonstrated to be reliable and valid among the Chinese population [45,46]. In this study, the Cronbach α of this instrument was 0.931, 0.892, 0.946, and 0.937, respectively, for the 4 assessments.

#### Secondary Outcomes

##### Fatigue

The Fatigue Scale-14 has been proven valid and reliable among the general population in China [47], which consists of 14 items, with each item rated on a 2-point scale (0=no and 1=yes). This scale is most commonly used and studied to assess the severity of fatigue over the past 2 weeks [48]. In this study, the sum of scores on each item was calculated, and higher total scores reflect higher levels of fatigue. The Cronbach α of the instrument was 0.802, 0.824, 0.868, and 0.857, respectively, for the 4 assessments.

##### Personality-Neuroticism

Personality-neuroticism was assessed by the NEO Five-Factor Inventory, which was developed by Costa and McCrae [49] in 1987 and adapted for the Chinese population by Jianxin Zhang. This instrument is sensitive to personality across neuroticism, and higher total scores indicate that an individual is more likely and easily to feel nervous and anxious. The Cronbach α of the instrument was 0.801, 0.868, 0.884, and 0.887, respectively, for the 4 assessments.

##### Quality of Sleep

Designed by Li et al [50], the Self-Rating Scale of Sleep was adapted to evaluate the quality of sleep among students in the past month. This instrument contains 10 items, with each item rated on a 5-point Likert scale ranging from 1 to 5, and higher total scores of Self-Rating Scale of Sleep indicate worse quality of sleep [51]. The Cronbach α of the instrument in this study was 0.660, 0.816, 0.794, and 0.795, respectively, for the 4 assessments.

##### Resilience

The 10-item Connor-Davidson Resilience Scale was used to measure participants’ psychological resilience, which was developed by Connor and Davidson [52] in 2003 with 25 items and was reduced to 10 items by Campbell-Sills and Stein in 2007 [53]. This instrument consists of 10 items rated on a 4-point Likert scale (ranging from 1 to 4) and has been validated among parents of children diagnosed with cancer and survivors of earthquakes in China [54,55]. Higher total scores on the 10-item Connor-Davidson Resilience Scale indicate better psychological resilience. The Cronbach α of the instrument in this study was 0.924, 0.944, 0.957, and 0.963, respectively, for the 4 assessments.

##### Mindfulness

The 5-item Mindful Awareness Attention Scale was developed to evaluate the mindfulness level by Brown and Ryan [56] in 2003 and was simplified to 5 items by Caycho-Rodríguez et al [57] in 2021. This instrument was translated into Chinese through a rigorous process and adapted in Chinese nursing students by our research team [58]. The 5-item Mindful Awareness Attention Scale contains 5 items, and each item was rated on a 7-point Likert scale (0=never to 6=always), with higher scores reflecting a lower level of mindfulness [59]. The Cronbach α of the instrument in this study was 0.829, 0.855, 0.898, and 0.893, respectively, for the 4 times.

Meanwhile, demographic information, including age, sex, ethnicity, education level, student cadre status, only-child status, annual family income, smoking and alcohol use, and reason for choosing a nursing major, was obtained using self-reported questionnaires. The acceptance of the neurofeedback-assisted web-based mindfulness intervention in the NAOM group—delivered via the FocusZen headband and the “OxyZen” app—was assessed using the Technology Acceptance Model across 4 dimensions: perceived ease of use, perceived usefulness, attitude, and behavioral intention [60]. In addition, EEG data of resting state with eyes closed and during meditation state of all participants was also recorded simultaneously in this study. The power spectra of theta (4-8 Hz), alpha (8-13 Hz), beta (13-30 Hz), and gamma (>30 Hz) frequency bands were also calculated.

### Statistical Analysis

We performed all analyses based on a modified intention-to-treat population that includes all randomized participants who received at least 1 session of the intervention [61]. Descriptive analyses were used to describe the demographic characteristics of the participants. One-way ANOVA, chi-square tests, and Fisher exact tests were used to examine the comparability of the baseline demographic variables and psychological measurements among the 3 groups. Generalized estimating equation (GEE) models were used to analyze intervention effects, as they are appropriate for longitudinal data with repeated measures and allow for the examination of both main effects and group-by-time interaction effects across multiple variables. Simple effects with multiple comparisons using the least significant difference method were used to evaluate the effect of intervention and time when the interaction effect was statistically significant. The simple effects of the group and time would be reassessed when the interaction effects were statistically significant. The significance level of this study was determined at α=.05. All statistical analyses were completed with SPSS Statistics (version 24.0; IBM Corp) and SAS (version 9.4; SAS institute).

## Results

### Demographic Characteristics

**Figure 5:**
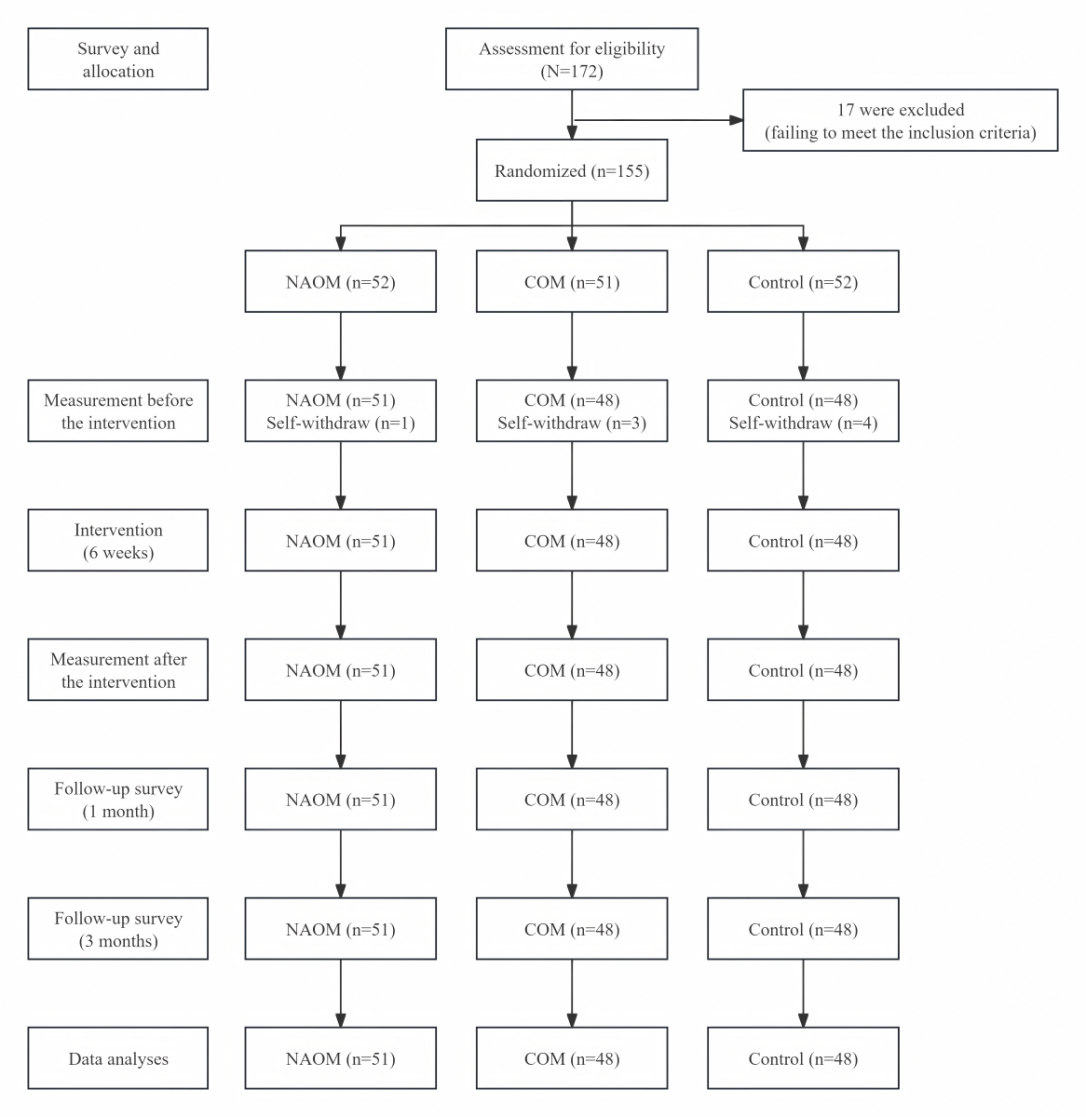
Flowchart of the 3-armed randomized controlled trial of web-based mindfulness interventions with or without neurofeedback assistance. Self-withdraw criteria were scheduling conflicts with academic obligations or clinical rotations. COM: conventional online mindfulness intervention; NAOM: neurofeedback-assisted online mindfulness intervention.

Of the 172 applicants screened for this study, 17 were deemed ineligible due to either unwillingness to participate in the intervention or failure to meet the inclusion criteria. A total of 155 participants were initially included and randomly allocated to 1 of the 3 groups: 52 in the NAOM group, 51 in the COM group, and 52 in the control group. After randomization and allocation, 1 participant in the NAOM group, 3 in the COM group, and 4 in the control group refused to continue. No more participants dropped out during the intervention and the follow-up assessments. As a result, of the 147 participants, 51 (98.08%) in the NAOM group, 48 (94.12%) in the COM group, and 48 (92.31%) in the control group completed all assessments and were included in the final analyses. The trial design and participant flow are summarized in Figure 5.

Table 2 summarizes the baseline demographic characteristics of participants across different groups. A total of 147 nursing students completed all assessments, with an average age of 20.44 (SD 3.06) years. Of the 147 students, 117 (79.59%) were female, 135 (91.84%) were of Han ethnicity, 128 (87.07%) were undergraduate students, 59 (40.14%) held positions as student cadres, 76 (51.7%) were the only child in their family, 65 (44.22%) had an annual family income of CNY ≤100,000, 109 (74.15%) chose the nursing major on their own will, 31 (21.09%) had family members engaged in nursing, and 21 (14.29%) have ever been diagnosed with mental illness such as anxiety and depression. There were no significant cross-group differences in demographic characteristics in this study.

**Table 2:** Demographic characteristics of participants across the 3 groups.

Variable			NAOM^a^ (n=51)		COM^b^ (n=48)		Control (n=48)		*F* test or chi-square test (*df*)			*P* value	
Age (y), mean (SD)			20.49 (1.69)		20.77 (1.68)		20.06 (4.79)		0.650 (2, 144)^c^			.52	
Sex n (%)										0.287 (2)^d^			.90
	Male	10 (33)		9 (30)		11 (37)							
	Female	41 (35)		39 (33)		37 (32)							
Ethnicity, n (%)										0.123 (2) ^d^			.99
	Han	47 (35)		44 (33)		44 (33)							
	Other	4 (33)		4 (33)		4 (33)							
Grade, n (%)										0.186 (2) ^d^			.91
	Undergraduate	45 (35)		41 (32)		42 (33)							
	Postgraduate	6 (32)		7 (37)		6 (32)							
Student cadre status, n (%)										0.805 (2) ^d^			.67
	No	33 (38)		28 (32)		27 (31)							
	Yes	18 (31)		20 (34)		21 (36)							
Only-child status, n (%)										3.344 (2) ^d^			.19
	No	27 (38)		18 (25)		26 (37)							
	Yes	24 (32)		30 (39)		22 (29)							
Family annual income (CNY), n (%)										1.856 (4) ^d^			.78
	<100,000 (US $13,880)	23 (35)		19 (29)		23 (35)							
	100,000-300,000 (US $13,880-$41,640)	23 (33)		26 (38)		20 (29)							
	≥300,000 (US $41,640)	5 (38)		3 (23)		5 (38)							
Smoking habits, n (%)										1.74 (2) ^d^			.77
	No	49 (34)		48 (33)		47 (33)							
	Yes	2 (67)		0 (0)		1 (33)							
Alcohol use in the past 6 months, n (%)										4.005 (2) ^d^			.36
	No	32 (34)		34 (36)		28 (30)							
	<3 times a week	19 (37)		13 (25)		20 (38)							
	3-5 times a week	0 (0)		1 (100)		0 (0)							
	≥5 times a week	0 (0)		0 (0)		0 (0)							
Reason for choosing a nursing major, n (%)										0.437 (2) ^d^			.80
	Not own will	12 (32)		12 (32)		14 (37)							
	Own will	39 (36)		36 (33)		34 (31)							
Have family members engaged in nursing, n (%)										1.933 (2) ^d^			.38
	No	43 (37)		38 (33)		35 (30)							
	Yes	8 (26)		10 (32)		13 (42)							
Have been diagnosed with mental illnesses such as anxiety and depression, n (%)										4.908 (6) ^d^			.57
	No	47 (37)		40 (32)		39 (31)							
	Anxiety	3 (25)		4 (33)		5 (42)							
	Depression	1 (13)		3 (38)		4 (50)							
	Others	0 (0)		1 (100)		0 (0)							
Depressive symptoms, mean (SD)			6.73 (4.05)		7.42 (5.01)		6.98 (4.53)		0.293 (2, 144) ^c^			.75	
Anxiety symptoms, mean (SD)			5.41 (3.72)		6.60 (4.75)		5.42 (4.25)		1.267 (2, 144) ^c^			.29	
Fatigue, mean (SD)			7.06 (3.37)		7.44 (3.68)		7.27 (3.62)		0.141 (2, 144) ^c^			.87	
Quality of sleep, mean (SD)			20.94 (4.14)		22.73 (4.62)		22.04 (4.91)		1.947 (2, 144) ^c^			.15	
Personality-neuroticism, mean (SD)			22.75 (8.45)		25.04 (7.83)		23.73 (7.41)		1.044 (2, 144) ^c^			.36	
Resilience, mean (SD)			29.02 (5.73)		27.63 (4.79)		29.21 (4.83)		1.364 (2, 144) ^c^			.26	
Mindfulness, mean (SD)			15.08 (4.97)		15.40 (4.79)		14.96 (4.36)		0.110 (2, 144) ^c^			.90	

^a^NAOM: neurofeedback-assisted online mindfulness intervention.

^b^COM: conventional online mindfulness intervention.

^c^*F* test.

^d^Chi-square test.

### GEE Analysis

The results of the GEE analysis are summarized in Table 3. The parameter estimation of GEE analysis showed that interaction effects of depressive symptoms, anxiety symptoms, fatigue, and mindfulness were statistically significant, with *P* values <.001. Meanwhile, neither the interaction (time×group) nor the main effect of group difference was found in the quality of sleep, personality-neuroticism, and resilience. The simple effects of groups and time are summarized in Tables 4 and 5.

**Table 3:** Results of the GEE^a^ analysis.

Variables			T1^b^, mean (SD)		T2^c^, mean (SD)		T3^d^, mean (SD)		T4^e^, mean (SD)		Time simple effect, *P* value		Main group, *P* value			Main time, *P* value			Interaction, *P* value	
Depressive symptoms														.09			<.001			<.001
	Group 1^f^	6.73 (4.05)		2.27 (2.69)^g^		5.24 (4.86)^g,h^		3.22 (4.04)^g,i^		<.001										
	Group 2^j^	7.42 (5.01)		3.73 (3.21)^g,k^		5.75 (4.45)^g,h^		5.25 (5.38)^g,h^		<.001										
	Group 3^l^	6.98 (4.53)		5.60 (4.36)^g,j,m^		5.54 (5)		4.67 (3.98)^g,h^		.001										
	Group simple effect, *P* value	.75		<.001		.86		.07		—^n^										
Anxiety symptoms														.001			<.001			<.001
	Group 1	5.41 (3.72)		1.82 (2)^g^		3.06 (3.37)^g,h^		1.55 (2.36)^g^^,i^		<.001										
	Group 2	6.60 (4.75)		3.54 (3.05)^g^^,k^		5.20 (4.68)^g,h,k^		3.58 (4.57)^g,i,k^		<.001										
	Group 3	5.42 (4.25)		5.29 (4.07)^k,m^		3.96 (4.23)^g,h^		3.12 (3.69)^g,h,k^		<.001										
	Group simple effect, *P* value	.29		<.001		.04		.005		—										
Fatigue														.16			<.001			.001
	Group 1	7.06 (3.37)		3.92 (3.24)^g^		4.96 (4.20)^g,h^		3.67 (3.45)^g,i^		<.001										
	Group 2	7.44 (3.68)		4.31 (3.26)^g^		5.54 (3.70)^g,h^		5.02 (3.77)^g^		<.001										
	Group 3	7.27 (3.62)		6.54 (3.81)^k,m^		5.21 (3.83)^g,h^		4.98 (3.76)^g,h^		<.001										
	Group simple effect, *P* value	.87		<.001		.76		.11		—										
Quality of sleep														.07			0.001			—
	Group 1	20.94 (4.14)		18.25 (4.27)^g^		17.45 (4.40)^g^		16.10 (4.15)^g,h,i^		<.001										
	Group 2	22.73 (4.62)		19.56 (4.91)^g^		18.79 (4.40)^g^		17.04 (4.08)^g,h,i^		<.001										
	Group 3	22.04 (4.91)		19.75 (4.91)^g^		19.85 (6.14)^g^		17.67 (5.64)^g,h,i^		<.001										
	Group simple effect, *P* value	.15		.31		.06		.25		—										
Personality-neuroticism														.19			<.001			—
	Group 1	22.75 (8.45)		17.82 (9.24)^g^		18.24 (9.87)^g^		15.76 (10.30)^g,h,i^		<.001										
	Group 2	25.04 (7.83)		19.48 (9.73)^g^		21.58 (10.30)^g,h^		19.04 (10.56)^g,i^		<.001										
	Group 3	23.73 (7.41)		19.65 (9.09)^g^		19.33 (9.56)^g^		18.85 (9.08)^g^		.02										
	Group simple effect, *P* value	.36		.56		.24		.19		—										
Resilience														.66			.002			—
	Group 1	29.02 (5.73)		30.76 (4.16)		31.25 (5.19)^g^		31.08 (6.17)		.20										
	Group 2	27.63 (4.79)		30.88 (5.63)^g^		30.27 (5.24)^g^		31.40 (5.66)^g^		0.003										
	Group 3	29.21 (4.83)		29.33 (5.91)		30.15 (6.61)		30.48 (5.8)		.18										
	Group simple effect, *P* value	.26		.28		.57		.74		—										
Mindfulness														<.001			<.001			<.001
	Group 1	15.08 (4.97)		7.90 (2.77)^g^		13.24 (5.52)^g^		12.33 (5.01)^g^^,h^		<.001										
	Group 2	15.40 (4.79)		11.67 (3.28)^k^^,m^		14.92 (4.61)		13.67 (4.86)^g,h,i^		<.001										
	Group 3	14.96 (4.36)		14.58 (4.69)^k^^,m^		15.23 (5.56)		14.58 (5.48)		.83										
	Group simple effect, *P* value	.90		<.001		.13		.09		—										

^a^GEE: generalized estimating equation.

^b^T1: baseline.

^c^T2: postintervention.

^d^T3: 1-month follow-up.

^e^T4: 3-month follow-up.

^f^Group 1: neurofeedback-assisted online mindfulness intervention group.

^g^Significant compared with baseline.

^h^Significant compared with postintervention.

^i^Significant compared with 1-month follow-up.

^j^Group 2: conventional online mindfulness intervention group

^k^Significant compared with group 1.

^l^Group 3: control group.

^m^Significant compared with group 2.

^n^Not applicable.

**Figure 6:**
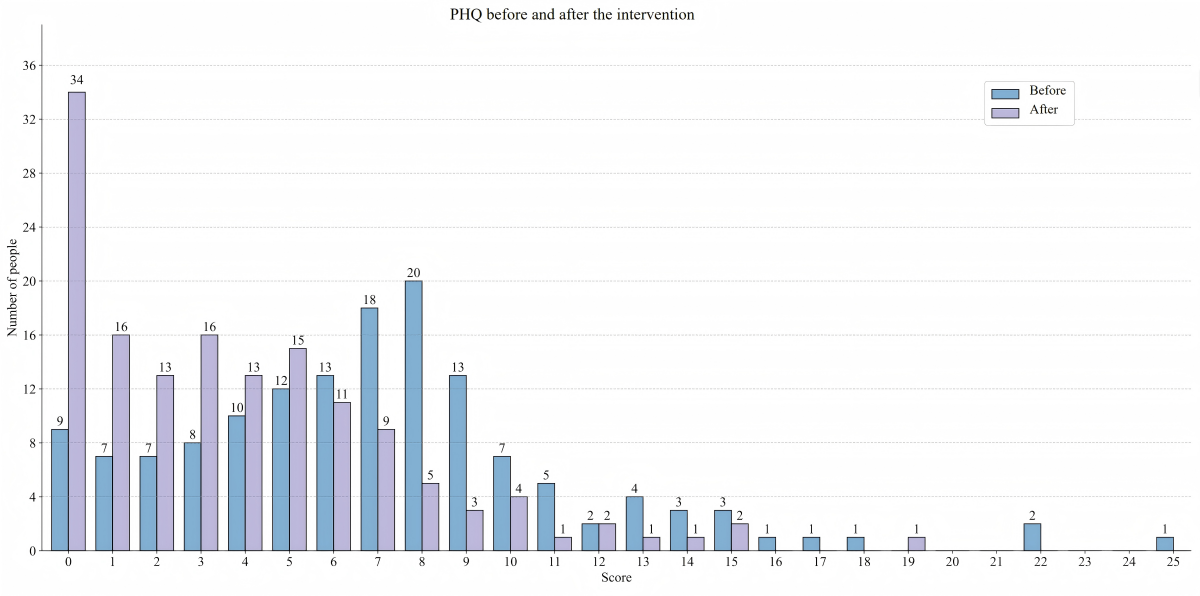
The number of participants and their scores on the 9-item Patient Health Questionnaire (PHQ-9) before and after the intervention.

**Figure 7:**
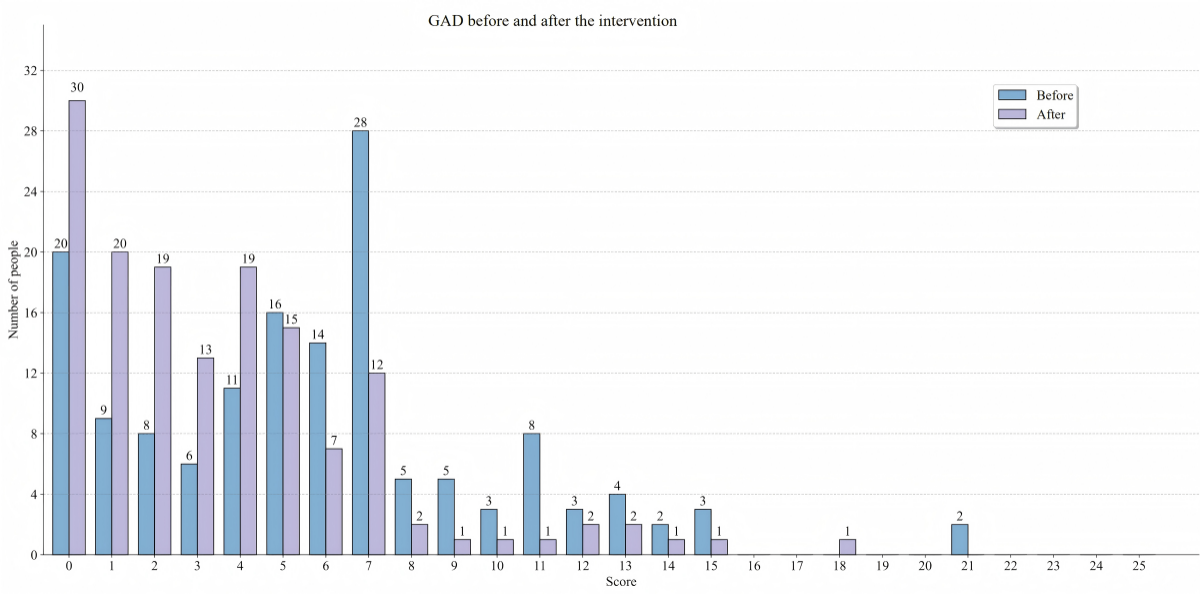
The number of participants and their scores on the Generalized Anxiety Disorder Questionnaire-7 (GAD-7) before and after the intervention.

The scores of PHQ-9 and GAD-7 in all 3 groups decreased at the postintervention assessment, and compared with before the intervention, the number of individuals with PHQ-9 and GAD-7 scoring 0 to 4 increased after the intervention, as shown in Figures 6 and 7. Significant reductions in symptoms of depression, anxiety, and fatigue were observed in the NAOM (MD=−3.330, Cohen *d*=0.926, *P*<.001; MD=−3.468, Cohen *d*=1.091, *P*<.001; MD=−2.620, Cohen *d*=0.743, *P*<.001, respectively) and COM (MD=−1.875, Cohen *d*=0.490, *P*=.03; MD=−1.750, Cohen *d*=0.486, *P*=.02; MD=−2.229, Cohen *d*=0.629, *P*=.01, respectively) groups compared with the control group at postintervention assessment. Meanwhile, the NAOM (MD=−6.681; Cohen *d*=1.750; *P*<.001) and COM (MD=−2.917; Cohen *d*=0.722; *P*<.001) groups showed better effects in improving the level of mindfulness. Moreover, significant differences in depressive symptoms (MD=−1.455; Cohen *d*=0.492; *P*=.04), anxiety symptoms (MD=−1.718; Cohen *d*=0.670; *P*=.04), and level of mindfulness (MD=−3.765; Cohen *d*=1.245; *P*<.001) were also found between the NAOM group and the COM group at the postintervention assessment. However, except for anxiety symptoms (MD=−1.576, Cohen *d*=0.512, *P*=.01; MD=−2.034, Cohen *d*=0.565, *P*=.02), there was no significant difference between the 3 groups at the 1- and 3-month follow-ups.

**Table 4:** Simple effects of groups.

Variables				MD^a^ (SE)		*df*		*P* value		Cohen *d*
Depressive symptoms										
	Baseline									
		Group 1^b^–group 3^c^	−0.254 (0.912)		144		.96		0.060	
		Group 2^d^–group 3	0.438 (0.925)		144		.89		0.092	
		Group 1–group 2	−0.691 (0.912)		144		.75		0.152	
	After intervention									
		Group 1–group 3	−3.330 (0.699)		144		<.001		0.926	
		Group 2–group 3	−1.875 (0.710)		144		.03		0.490	
		Group 1–group 2	−1.455 (0.699)		144		.04		0.492	
	1-month follow-up									
		Group 1–group 3	−0.306 (0.960)		144		.95		0.062	
		Group 2–group 3	0.208 (0.975)		144		.98		0.044	
		Group 1–group 2	−0.515 (0.960)		144		.85		0.110	
	3-month follow-up									
		Group 1–group 3	−1.451 (0.905)		144		.08		0.362	
		Group 2–group 3	0.583 (0.919)		144		.80		0.123	
		Group 1–group 2	−2.034 (0.905)		144		.08		0.430	
Anxiety symptoms										
	Baseline									
		Group 1–group 3	−0.005 (0.855)		144		.99		0.001	
		Group 2–group 3	1.188 (0.868)		144		.52		0.263	
		Group 1–group 2	−1.192 (0.855)		144		.50		0.281	
	After intervention									
		Group 1–group 3	−3.468 (0.631)		144		<.001		1.091	
		Group 2–group 3	−1.750 (0.640)		144		.02		0.486	
		Group 1–group 2	−1.718 (0.631)		144		.02		0.670	
	1-month follow-up									
		Group 1–group 3	−0.900 (0.827)		144		.52		0.236	
		Group 2–group 3	1.229 (0.839)		144		.31		0.276	
		Group 1–group 2	−2.129 (0.827)		144		.03		0.525	
	3-month follow-up									
		Group 1–group 3	−1.576 (0.730)		144		.01		0.512	
		Group 2–group 3	0.458 (0.741)		144		.81		0.110	
		Group 1–group 2	−2.034 (0.730)		144		.02		0.565	
Fatigue										
	Baseline									
		Group 1–group 3	−0.212 (0.715)		144		.95		0.061	
		Group 2–group 3	0.167 (0.726)		144		.97		0.046	
		Group 1–group 2	−0.379 (0.715)		144		.86		0.107	
	After intervention									
		Group 1–group 3	−2.620 (0.692)		144		<.001		0.743	
		Group 2–group 3	−2.229 (0.703)		144		.01		0.629	
		Group 1–group 2	−0.391 (0.692)		144		.84		0.120	
	1-month follow-up									
		Group 1–group 3	−0.248 (0.789)		144		.95		0.062	
		Group 2–group 3	0.333 (0.801)		144		.91		0.089	
		Group 1–group 2	−0.581 (0.789)		144		.74		0.147	
	3-month follow-up									
		Group 1–group 3	−1.313 (0.735)		144		.18		0.364	
		Group 2–group 3	0.042 (0.746)		144		.99		0.011	
		Group 1–group 2	−1.354 (0.735)		144		.16		0.375	
Mindfulness										
	Baseline									
		Group 1–group 3	0.120 (0.949)		144		.99		0.026	
		Group 2–group 3	0.438 (0.963)		144		.89		0.096	
		Group 1–group 2	−0.317 (0.949)		144		.94		0.065	
	After intervention									
		Group 1–group 3	−6.681 (0.734)		144		<.001		1.750	
		Group 2–group 3	−2.917 (0.745)		144		<.001		0.722	
		Group 1–group 2	−3.765 (0.734)		144		<.001		1.245	
	1-month follow-up									
		Group 1–group 3	−1.994 (1.060)		144		.15		0.360	
		Group 2–group 3	−0.313 (1.070)		144		.95		0.061	
		Group 1–group 2	−1.681 (1.060)		144		.25		0.330	
	3-month follow-up									
		Group 1–group 3	−2.250 (1.030)		144		.08		0.429	
		Group 2–group 3	−0.917 (1.050)		144		.66		0.177	
		Group 1–group 2	−1.333 (1.030)		144		.40		0.270	

^a^MD: mean difference.

^b^Group 1: neurofeedback-assisted online mindfulness intervention group.

^c^Group 3: control group.

^d^Group 2: conventional online mindfulness intervention group.

**Table 5:** Simple effects of time.

Variables				MD^a^ (SE)		*df*		*P* value		Generalized η^2^	
Depressive symptoms											
	Group 1^b^										0.162
		T2^c^-T1^d^	−4.451 (0.605)		50		<.001				
		T3^e^-T1	−1.490 (0.572)		50		.02				
		T4^f^-T1	−3.510 (0.485)		50		<.001				
		T3-T2	2.961 (0.710)		50		<.001				
		T4-T2	0.941 (0.580)		50		.38				
		T4-T3	−2.020 (0.430)		50		<.001				
	Group 2^g^										0.078
		T2-T1	−3.688 (0.662)		47		<.001				
		T3-T1	−1.667 (0.610)		47		.04				
		T4-T1	−2.167 (0.576)		47		.003				
		T3-T2	2.021 (0.563)		47		.004				
		T4-T2	1.521 (0.667)		47		.03				
		T4-T3	−0.500 (0.490)		47		.74				
	Group 3^h^										0.034
		T2-T1	−1.375 (0.579)		47		.02				
		T3-T1	−1.438 (0.754)		47		.06				
		T4-T1	−2.313 (0.620)		47		<.001				
		T3-T2	−0.063 (0.405)		47		.88				
		T4-T2	−0.938 (0.360)		47		.01				
		T4-T3	−0.875 (0.463)		47		.07				
Anxiety symptoms											
	Group 1										0.215
		T2-T1	−3.588 (0.529)		50		<.001				
		T3-T1	−2.353 (0.556)		50		<.001				
		T4-T1	−3.863 (0.482)		50		<.001				
		T3-T2	1.235 (0.364)		50		.01				
		T4-T2	−0.275 (0.291)		50		.78				
		T4-T3	−1.510 (0.389)		50		.002				
	Group 2										0.081
		T2-T1	−3.063 (0.793)		47		<.001				
		T3-T1	−1.417 (0.646)		47		.03				
		T4-T1	−3.021 (0.527)		47		<.001				
		T3-T2	1.646 (0.661)		47		.02				
		T4-T2	0.042 (0.753)		47		.96				
		T4-T3	−1.604 (0.584)		47		.01				
	Group 3										0.053
		T2-T1	−0.125 (0.626)		47		.84				
		T3-T1	−1.458 (0.607)		47		.02				
		T4-T1	−2.292 (0.555)		47		<.001				
		T3-T2	−1.333 (0.461)		47		.01				
		T4-T2	−2.167 (0.481)		47		<.001				
		T4-T3	−0.833 (0.490)		47		.10				
Fatigue											
	Group 1										0.124
		T2-T1	−3.137 (0.465)		50		<.001				
		T3-T1	−2.098 (0.628)		50		.01				
		T4-T1	−3.392 (0.505)		50		<.001				
		T3-T2	1.039 (0.480)		50		.04				
		T4-T2	−0.255 (0.485)		50		.95				
		T4-T3	−1.294 (0.430)		50		.02				
	Group 2										0.095
		T2-T1	−3.125 (0.444)		47		<.001				
		T3-T1	−1.896 (0.532)		47		.01				
		T4-T1	−2.417 (0.524)		47		<.001				
		T3-T2	1.229 (0.440)		47		.04				
		T4-T2	0.708 (0.535)		47		.55				
		T4-T3	−0.521 (0.541)		47		.77				
	Group 3										0.061
		T2-T1	−0.729 (0.526)		47		.17				
		T3-T1	−2.062 (0.511)		47		<.001				
		T4-T1	−2.292 (0.456)		47		<.001				
		T3-T2	−1.333 (0.587)		47		.03				
		T4-T2	−1.562 (0.549)		47		.01				
		T4-T3	−0.229 (0.537)		47		.67				
Mindfulness											
	Group 1										0.244
		T2-T1	−7.176 (0.784)		50		<.001				
		T3-T1	−1.843 (1.140)		50		.38				
		T4-T1	−2.745 (0.911)		50		.02				
		T3-T2	5.333 (0.888)		50		<.001				
		T4-T2	4.431 (0.851)		50		<.001				
		T4-T3	−0.902 (0.790)		50		.67				
	Group 2										0.098
		T2-T1	−3.729 (0.802)		47		<.001				
		T3-T1	−0.479 (0.887)		47		.59				
		T4-T1	−1.729 (0.837)		47		.04				
		T3-T2	3.250 (0.777)		47		<.001				
		T4-T2	2.000 (0.727)		47		.01				
		T4-T3	−1.250 (0.635)		47		.05				
	Group 3										0.003
		T2-T1	−0.375 (0.919)		47		.98				
		T3-T1	0.271 (1.049)		47		.99				
		T4-T1	−0.375 (0.950)		47		.98				
		T3-T2	0.646 (0.736)		47		.82				
		T4-T2	<0.001 (0.678)		47		.99				
		T4-T3	−0.646 (0.908)		47		.89				

^a^MD: mean difference.

^b^Group 1: neurofeedback-assisted online mindfulness intervention group.

^c^T2: postintervention.

^d^T1: baseline.

^e^T3: 1-month follow-up.

^f^T4: 3-month follow-up.

^g^Group 2: conventional online mindfulness intervention group.

^h^Group 3: control group.

## Discussion

### Principal Findings

This 3-armed randomized controlled trial examined the effectiveness of a 6-week web-based mindfulness program in enhancing the mental health of nursing students in China and explored the ability of neurofeedback technology to enhance the effectiveness of the conventional web-based mindfulness intervention. Compared with the control group after the intervention, the 6-week web-based mindfulness intervention, both the neurofeedback-assisted and conventional 6-week web-based mindfulness interventions led to significant reductions in depression, anxiety, and fatigue, along with increased mindfulness levels. Meanwhile, the results of this study indicated that the neurofeedback-assisted web-based mindfulness intervention was more effective than the conventional mindfulness intervention in alleviating mental health problems in the short term after the intervention, and it also had a positive effect on the long-term relief of anxiety symptoms. However, no other statistically significant differences between the NAOM group and the COM group were observed in the long term, indicating that the neurofeedback-assisted web-based mindfulness intervention has limited advantage in improving long-term mental health status in this study.

Web-based mindfulness interventions are effective in improving some of the common mental health problems [19]. Our findings align with previous studies showing that web-based mindfulness interventions successfully decreased symptoms of anxiety, depression, and fatigue among the Chinese nursing students [62-64], but this study also adds to the literature by demonstrating the effectiveness of combining mindfulness with a neurofeedback intervention in the short term. Findings of this study are similar to a previous study conducted in South Korea, which found that 8 weeks of mindfulness practice reduces symptoms of anxiety and depression in nursing students [65]. However, our study also shows the effectiveness of web-based and short-term interventions, which overcome issues of accessibility and time constraints. In addition, web-based mindfulness has been proven to be cost-effective; it can effectively alleviate mental problems and prevent symptom exacerbation, and hence, it significantly reduces the health care costs [66,67]. Cost-effectiveness analysis of web-based mindfulness interventions conducted among nursing students also demonstrated good economic benefits; it had a positive impact on increasing academic success, decreasing attrition, and promoting occupational sustainability among nursing students, thereby mitigating nursing workforce shortages [25,68]. Meanwhile, none of the participants in the NAOM group and the COM group dropped out after the beginning of the mindfulness intervention, demonstrating good adherence among the nursing students and supporting the feasibility of offering web-based mindfulness interventions to this population. In addition, video- and audio-based web-based mindfulness interventions are cost-effective, substantially reducing labor costs and enabling large-scale solution deployment. Therefore, nursing education providers could consider incorporating web-based mindfulness interventions into the curriculum for nursing students to maintain or improve their psychological well-being by supporting the prevention and management of anxiety, depression, and fatigue symptoms.

The changes in brain activity and biomarkers of immune function and stress provide a neurophysiological basis for explaining the positive effects of mindfulness interventions. Various studies in neuroscience have confirmed the biological basis of mindfulness-based interventions in alleviating depression and anxiety. Specifically, mindfulness-based interventions could decrease individuals’ levels of interleukin-6 and tumor necrosis factor alpha, which could lower the risk of mental health problems and improve physical health [69,70]. Another 4-week mindfulness intervention study collected EEG data from participants and found that the power of alpha waves significantly increased during mindfulness practice, which is closely related to the relaxation state [71]. Meanwhile, neuroimaging studies have demonstrated that mindfulness-based interventions could activate the prefrontal cortex function, which could modulate the emotion-generative system and improve emotion regulation [72]. A pilot randomized controlled trial involving 82 healthy university students found that participants who received web-based mindfulness interventions showed significantly greater functional near-infrared spectroscopy signal activation in the prefrontal cortex compared to those in the control group, indicating that continuous mindfulness-based intervention could alter prefrontal cortex function and revealing the mechanism of the web-based mindfulness intervention [73]. In the future, web-based mindfulness interventions are supposed to be applied in patients with somatic diseases to further verify their effectiveness in different populations, such as patients with cancer and patients with chronic diseases (diabetes, hypertension, or chronic obstructive pulmonary disease).

Our findings indicated that the NAOM demonstrated long-term effects on alleviating anxiety symptoms at both the 1-month and 3-month follow-ups, whereas no significant differences were observed in the assessments of other outcomes. Interventional studies in patients with generalized anxiety disorder and healthy participants have revealed that the NAOM effectively enhanced the alpha wave activity and modulated frontal alpha asymmetry [36]. Alpha waves, recognized as the dominant EEG rhythm during resting states in healthy adults, are associated with calm and relaxed states [74]. While conventional mindfulness interventions have also been shown to increase the average alpha wave power across multiple brain regions, neurofeedback, which used computer-assisted technology to train patients in recalibrating maladaptive brain wave patterns, was considered to further augment the effects of mindfulness interventions [75,76]. Nevertheless, current research on NAOM remains scarce, and its long-term effects on reducing anxiety, depression, and fatigue symptoms require further investigation.. Additional studies are warranted to validate these findings and elucidate the underlying neural mechanisms. In addition, controversy also remains regarding the long-term effects of mindfulness interventions [77-79]. It is noteworthy that compared to general mental health education, the 6-week web-based mindfulness intervention did not exhibit statistically significant long-term effects based on the results of follow-up assessments. Similar results have been reported in previous studies on other populations. A study using a mindfulness-based stress reduction program conducted among medical students in Saudi Arabia found that the mindfulness intervention had a positive effect on the mental well-being of medical students, but the differences between the intervention and control groups were insignificant in follow-up assessments, except for anxiety symptoms [80]. A meta-analysis also demonstrated that the benefits of mindfulness interventions could be observable right after starting to practice, but these effects tended to diminish over time without continuous practice [81]. However, some other studies have reported the long-term effectiveness of mindfulness in alleviating mental problems in nursing students and other populations [77-79]. Future studies should include longitudinal follow-ups of mindfulness interventions for nursing students to clarify their long-term effects on mental health, career choices, clinical care competencies, and patient outcomes. Meanwhile, considering that short-term mindfulness practice might not be robust enough to ensure lasting effects based on existing evidence, we suggested that educators could encourage nursing students to practice mindfulness regularly and incorporate web-based mindfulness into regular courses or extracurricular routine activities.

In the alleviation of mental health problems, the NAOM group showed ongoing improvements compared with the baseline assessment, with significant differences from not only the control group but also the COM group at the immediate postintervention assessment, which proved the effects of neurofeedback technology in assisting web-based mindfulness interventions. A systematic review demonstrated that neurofeedback technology could help participants practice mindfulness correctly and lead to better attention, deeper mindfulness, and positive behavioral outcomes [82]. A randomized controlled trial conducted among employees in South Korea analyzed the effectiveness of a neurofeedback-assisted web-based mindfulness training program in alleviating stress and mental health problems and found that neurofeedback-assisted mindfulness training achieved superior outcomes in stress reduction and improvement of other psychological indices compared with web-based mindfulness training without neurofeedback, with effects persisting at the 1-month follow-up assessment [71]. A study involving 17 Chinese patients with anxiety disorders explored the effects of a neurofeedback system based on alpha band oscillation of EEG signals and found that mindfulness interventions with neurofeedback could have a positive effect on the brain activity pattern of patients with anxiety disorders and alleviate their anxiety symptoms [36].

Furthermore, we used postsession neurofeedback rather than traditional real-time neurofeedback in this study. A primary reason for this designation is that, unlike mindfulness meditation practice guided by white noise whose volume, speed, and content can be adjusted at any time [83], the mindfulness meditation practice used in this study was guided by instructional audios with fixed speed and content, on which the participants should always focus their attention during the practice, and real-time neurofeedback may distract their attention. Another practical reason is that the postsession neurofeedback could be more cost-effective, easier to integrate with various mindfulness intervention programs, and more convenient for large-scale promotion in the future, as it requires no costly equipment, immediate data processing, or artificial intelligence algorithms needed for real-time neurofeedback. However, few studies have rigorously evaluated the effectiveness of postsession neurofeedback, and further mechanism studies may be required to confirm its effectiveness. Meanwhile, we also did not use functional magnetic resonance imaging (fMRI) neurofeedback to assist the mindfulness intervention, although it could allow researchers to track and modulate brain function and has been proven effective in reducing mental health problems [84]. The reason is that fMRI neurofeedback is typically used in a single-time mindfulness intervention. This study requires multiple sessions of neurofeedback, and repeated fMRI acquisitions would significantly increase costs and potentially raise the dropout rate among participants.

This study has several limitations that suggest directions for future research. First, the participants in the study were self-selected from a single medical college in Beijing, China, who were predominantly young, female, and technology-savvy college nursing students, limiting the generalizability of our results to the whole nursing student population in China and to wider and more diverse populations. Therefore, future studies should be conducted among nursing students from a broader range of medical colleges in China, wider and more diverse populations, and patients with comorbidities of physical and mental illnesses. Second, the relatively small sample size might reduce the statistical power of the results, and future studies with a larger sample size are expected. Third, anxiety, depression, and other symptoms were assessed using self-reported measures, which might have been influenced by social desirability bias. Objective indicators such as electroencephalography and fMRI should be considered for less biased evaluation of the intervention effects in the future. Fourth, as this study was entirely quantitative, future research should incorporate qualitative methods to gain deeper insights into nursing students’ experiences with the interventions. Fifth, the participants were not asked to report if they had done any extra practice outside of the required lessons, which might be associated with intervention outcomes [85]. Sixth, cost-effectiveness estimates were not conducted in this study due to data acquisition constraints, which should be explored as a priority in future research. Finally, some possible mediators of the intervention effects were not investigated, such as mindfulness skills or rumination of the participants.

### Conclusions

The results of this randomized controlled trial provide initial evidence to support the benefits of the neurofeedback-assisted web-based mindfulness intervention and pioneer portable neurofeedback in web-based mindfulness, warranting larger, multisite trials. In summary, this study found that both NAOM and COM interventions were beneficial for nursing students in the short term, but the effects were not maintained over time. The neurofeedback component had a small incremental effect on reducing symptoms of anxiety and depression. Web-based mindfulness interventions have significant potential in alleviating mental health problems among nursing students, given this form aligns with the preferences of nursing students, reduces the perceived stigma associated with treatment seeking, and is readily scalable even for those living in areas with limited or no access to mental health services. Therefore, future research should explore the long-term effects of web-based mindfulness interventions, both conventional and neurofeedback assisted, in alleviating mental health problems and examining the underlying neural mechanisms in diverse samples of participants.

## Data Availability

The datasets generated or analyzed during this study are available from the corresponding author on reasonable request.

## Appendix

Multimedia Appendix 1 CONSORT-eHEALTH checklist (V 1.6.1).

## Abbreviations

COM: conventional online mindfulness intervention
EEG: electroencephalogram
fMRI: functional magnetic resonance imaging
GAD-7: Generalized Anxiety Disorder Questionnaire
GEE: generalized estimating equation
MD: mean difference
NAOM: neurofeedback-assisted online mindfulness intervention
PHQ-9: 9-item Patient Health Questionnaire
PUMCQS: Peking Union Medical College Questionnaire Survey
