# Correction: Effectiveness of Neurofeedback-Assisted and Conventional 6-Week Web-Based Mindfulness Interventions on Mental Health of Chinese Nursing Students: Randomized Controlled Trial

**DOI:** 10.2196/78147

**Published:** 2025-06-02

**Authors:** Shu Jing, Zhenwei Dai, Xiaoyang Liu, Xuelin Yang, Jinglei Cheng, Tianming Chen, Zihang Feng, Xin Liu, Fenghe Dong, You Xin, Zhuoyan Han, Haiyan Hu, Xiaoyou Su, Chen Wang

**Affiliations:** 1 School of Population Medicine and Public Health Chinese Academy of Medical Sciences & Peking Union Medical College Beijing China; 2 Peking University Sixth Hospital Peking University Beijing China; 3 Peking University Institute of Mental Health Peking University Beijing China; 4 National Health Commission Key Laboratory of Mental Health Peking University Beijing China; 5 National Clinical Research Center for Mental Disorders Peking University Sixth Hospital Beijing China; 6 School of Nursing and Institute of Nursing Research School of Medicine Zhejiang University Hangzhou China; 7 School of Nursing Chinese Academy of Medical Sciences & Peking Union Medical College Beijing China; 8 College of Computer Science and Technology Zhejiang University Hangzhou China; 9 The State Key Laboratory of Brain-machine Intelligence Zhejiang University Hangzhou China; 10 School of Translation and Interpreting Beijing Language and Culture University Beijing China; 11 Shenzhen Maternity and Child Healthcare Hospital Southern Medical University Shenzhen China; 12 State Key Laboratory of Respiratory Health and Multimorbidity Beijing China

In “Effectiveness of Neurofeedback-Assisted and Conventional 6-Week Web-Based Mindfulness Interventions on Mental Health of Chinese Nursing Students: Randomized Controlled Trial” (J Med Internet Res 2025;27: e71741.) the authors made fourteen clarifications.

The degree list for author Shu Jing was previously reported as:

PhD

And has now been changed to:

BSc.

The degree list for author Xiaoyang Liu was previously reported as:

MPH

And has now been changed to:

BA.

The degree list for author Xuelin Yang was previously reported as:

MPH.

And has now been changed to:

BSc.

The degree list for author Xin Liu was previously reported as:

MPH.

And has now been changed to:

BM.

The degree list for author Fenghe Dong was previously reported as:

PhD

And has now been changed to:

BE.

The degree list for author You Xin was previously reported as:

MPH

And has now been changed to:

BA.

The degree list for author Haiyan Hu was previously reported as:

Prof Dr Med

And has now been changed to:

MD.

The degree list for author Xiaoyou Su was previously reported as:

Prof Dr

And has now been changed to:

PhD.

The degree list for author Chen Wang and the Corresponding Author’s degree were previously reported as:

Prof Dr Med

and

Corresponding Author: Chen Wang, Prof Dr Med

And have now been changed to:

MD

and

Corresponding Author: Chen Wang, MD.

Additionally, the degrees of authors Jinglei Cheng, Tianming Chen, Zihang Feng, and Zhuoyan Han have been deleted since they are undergraduate students.

The correction will appear in the online version of the paper on the JMIR Publications website, together with the publication of this correction notice. Because this was made after submission to PubMed, PubMed Central, and other full-text repositories, the corrected article has also been resubmitted to those repositories.

