# Supplementary material for: Effectiveness of Neurofeedback-Assisted and Conventional 6-Week Web-Based Mindfulness Interventions on Mental Health of Chinese Nursing Students: Randomized Controlled Trial
Source: J Med Internet Res. 2025 May 23;27:e71741. doi: 10.2196/71741 (PMC12144476; doi:10.2196/71741)
Supplement: Multimedia Appendix 1 [file jmir_v27i1e71741_app1.pdf]

CONSORT-EHEALTH (V 1.6.1) - Submission/Publication Form

The CONSORT-EHEALTH checklist is intended for authors of randomized trials evaluating web-based and Internet-based applications/interventions, including mobile interventions, electronic games (incl multiplayer games), social media, certain telehealth applications, and other interactive and/or networked electronic applications. Some of the items (e.g. all subitems under item 5 - description of the intervention) may also be applicable for other study designs.

The goal of the CONSORT EHEALTH checklist and guideline is to be
a) a guide for reporting for authors of RCTs,
b) to form a basis for appraisal of an ehealth trial (in terms of validity)

CONSORT-EHEALTH items/subitems are MANDATORY reporting items for studies published in the Journal of Medical Internet Research and other journals / scientific societies endorsing the checklist.

Items numbered 1., 2., 3., 4a., 4b etc are original CONSORT or CONSORT-NPT (non-pharmacologic treatment) items.
Items with Roman numerals (i., ii, iii, iv etc.) are CONSORT-EHEALTH extensions/clarifications.

As the CONSORT-EHEALTH checklist is still considered in a formative stage, we would ask that you also RATE ON A SCALE OF 1-5 how important/useful you feel each item is FOR THE PURPOSE OF THE CHECKLIST and reporting guideline (optional).

Mandatory reporting items are marked with a red *.
In the textboxes, either copy & paste the relevant sections from your manuscript into this form - please include any quotes from your manuscript in QUOTATION MARKS,
or answer directly by providing additional information not in the manuscript, or elaborating on why the item was not relevant for this study.

YOUR ANSWERS WILL BE PUBLISHED AS A SUPPLEMENTARY FILE TO YOUR PUBLICATION IN JMIR AND ARE CONSIDERED PART OF YOUR PUBLICATION (IF ACCEPTED).
Please fill in these questions diligently. Information will not be copyedited, so please use proper spelling and grammar, use correct capitalization, and avoid abbreviations.

DO NOT FORGET TO SAVE AS PDF _AND_ CLICK THE SUBMIT BUTTON SO YOUR ANSWERS ARE IN OUR DATABASE !!!

Citation Suggestion (if you append the pdf as Appendix we suggest to cite this paper in the caption):
Eysenbach G, CONSORT-EHEALTH Group
CONSORT-EHEALTH: Improving and Standardizing Evaluation Reports of Web-based and Mobile Health Interventions
J Med Internet Res 2011;13(4):e126

URL: http://www.jmir.org/2011/4/e126/
doi: 10.2196/jmir.1923
PMID: 22209829

登录 Google 即可保存进度。了解详情

* 表示必填

Your name *
First Last

Shu Jing

Primary Affiliation (short), City, Country *
University of Toronto, Toronto, Canada

Chinese Academy of Medical Sciences & Pekir

Your e-mail address *
abc@gmail.com

pumcjs@sina.com

Title of your manuscript *
Provide the (draft) title of your manuscript.

Effectiveness of the neurofeedback-assisted and the conventional 6-week online mindfulness interventions on mental health of nursing students: a randomized controlled trial

Name of your App/Software/Intervention *

If there is a short and a long/alternate name, write the short name first and add the long name in brackets.

PUMCQS (PUMC Questionnaire Survey Applet)

Evaluated Version (if any)

e.g. "V1", "Release 2017-03-01", "Version 2.0.27913"

您的回答

Language(s) *

What language is the intervention/app in? If multiple languages are available, separate by comma (e.g. "English, French")

Chinese

URL of your Intervention Website or App

e.g. a direct link to the mobile app on app in appstore (itunes, Google Play), or URL of the website. If the intervention is a DVD or hardware, you can also link to an Amazon page.

您的回答

URL of an image/screenshot (optional)

您的回答

Accessibility *

Can an enduser access the intervention presently?

- ○ access is free and open
- ○ access only for special usergroups, not open
- ○ access is open to everyone, but requires payment/subscription/in-app purchases
- ◉ app/intervention no longer accessible
- ○ 其他:

Primary Medical Indication/Disease/Condition *

e.g. "Stress", "Diabetes", or define the target group in brackets after the condition, e.g. "Autism (Parents of children with)", "Alzheimers (Informal Caregivers of)"

Anxiety Symptoms Or Depression symptoms

Primary Outcomes measured in trial *

comma-separated list of primary outcomes reported in the trial

Anxiety symptoms measured by GAD-7, Depres

Secondary/other outcomes

Are there any other outcomes the intervention is expected to affect?

您的回答

Recommended "Dose" *

What do the instructions for users say on how often the app should be used?

- ○ Approximately Daily
- ○ Approximately Weekly
- ○ Approximately Monthly
- ○ Approximately Yearly
- ○ "as needed"
- ● 其他: Twice a week

Approx. Percentage of Users (starters) still using the app as recommended after 3 months *

- ○ unknown / not evaluated
- ○ 0-10%
- ○ 11-20%
- ○ 21-30%
- ○ 31-40%
- ○ 41-50%
- ○ 51-60%
- ○ 61-70%
- ○ 71%-80%
- ○ 81-90%
- ○ 91-100%
- ● 其他: All participants only used the applet for one and a half months， and 9[illegible]

Overall, was the app/intervention effective? *

- (selected) yes: all primary outcomes were significantly better in intervention group vs control
- partly: SOME primary outcomes were significantly better in intervention group vs control
- no statistically significant difference between control and intervention
- potentially harmful: control was significantly better than intervention in one or more outcomes
- inconclusive: more research is needed
- 其他:

Article Preparation Status/Stage *

At which stage in your article preparation are you currently (at the time you fill in this form)

- not submitted yet - in early draft status
- not submitted yet - in late draft status, just before submission
- (selected) submitted to a journal but not reviewed yet
- submitted to a journal and after receiving initial reviewer comments
- submitted to a journal and accepted, but not published yet
- published
- 其他:

Journal *

If you already know where you will submit this paper (or if it is already submitted), please provide the journal name (if it is not JMIR, provide the journal name under "other")

- ○ not submitted yet / unclear where I will submit this
- ◉ Journal of Medical Internet Research (JMIR)
- ○ JMIR mHealth and UHealth
- ○ JMIR Serious Games
- ○ JMIR Mental Health
- ○ JMIR Public Health
- ○ JMIR Formative Research
- ○ Other JMIR sister journal
- ○ 其他:

Is this a full powered effectiveness trial or a pilot/feasibility trial? *

- ○ Pilot/feasibility
- ◉ Fully powered

Manuscript tracking number *

If this is a JMIR submission, please provide the manuscript tracking number under "other" (The ms tracking number can be found in the submission acknowledgement email, or when you login as author in JMIR. If the paper is already published in JMIR, then the ms tracking number is the four-digit number at the end of the DOI, to be found at the bottom of each published article in JMIR)

- ○ no ms number (yet) / not (yet) submitted to / published in JMIR
- ◉ 其他: JMIR ms#71741

TITLE AND ABSTRACT

1a) TITLE: Identification as a randomized trial in the title

1a) Does your paper address CONSORT item 1a? *

I.e does the title contain the phrase "Randomized Controlled Trial"? (if not, explain the reason under "other")

- (●) yes
- ( ) 其他:

1a-i) Identify the mode of delivery in the title

Identify the mode of delivery. Preferably use “web-based” and/or “mobile” and/or “electronic game” in the title. Avoid ambiguous terms like “online”, “virtual”, “interactive”. Use “Internet-based” only if Intervention includes non-web-based Internet components (e.g. email), use “computer-based” or “electronic” only if offline products are used. Use “virtual” only in the context of “virtual reality” (3-D worlds). Use “online” only in the context of “online support groups”. Complement or substitute product names with broader terms for the class of products (such as “mobile” or “smart phone” instead of “iphone”), especially if the application runs on different platforms.

	1	2	3	4	5	
subitem not at all important	○	○	○	○	○	essential

Does your paper address subitem 1a-i? *

Copy and paste relevant sections from manuscript title (include quotes in quotation marks "like this" to indicate direct quotes from your manuscript), or elaborate on this item by providing additional information not in the ms, or briefly explain why the item is not applicable/relevant for your study

Used "online mindfulness interventions" in the title, as: Effectiveness of the neurofeedback-assisted and the conventional 6-week online mindfulness interventions on mental health of nursing students: a randomized controlled trial

1a-ii) Non-web-based components or important co-interventions in title

Mention non-web-based components or important co-interventions in title, if any (e.g., “with telephone support”).

	1	2	3	4	5	
subitem not at all important	○	○	○	○	○	essential

Does your paper address subitem 1a-ii?

Copy and paste relevant sections from manuscript title (include quotes in quotation marks "like this" to indicate direct quotes from your manuscript), or elaborate on this item by providing additional information not in the ms, or briefly explain why the item is not applicable/relevant for your study

您的回答

1a-iii) Primary condition or target group in the title

Mention primary condition or target group in the title, if any (e.g., “for children with Type I Diabetes”) Example: A Web-based and Mobile Intervention with Telephone Support for Children with Type I Diabetes: Randomized Controlled Trial

	1	2	3	4	5	
subitem not at all important	○	○	○	○	○	essential

Does your paper address subitem 1a-iii? *

Copy and paste relevant sections from manuscript title (include quotes in quotation marks "like this" to indicate direct quotes from your manuscript), or elaborate on this item by providing additional information not in the ms, or briefly explain why the item is not applicable/relevant for your study

Used "nursing students" in the title, as: Effectiveness of the neurofeedback-assisted and the conventional 6-week online mindfulness interventions on mental health of nursing students: a randomized controlled trial

1b) ABSTRACT: Structured summary of trial design, methods, results, and conclusions

NPT extension: Description of experimental treatment, comparator, care providers, centers, and blinding status.

1b-i) Key features/functionalities/components of the intervention and comparator in the METHODS section of the ABSTRACT

Mention key features/functionalities/components of the intervention and comparator in the abstract. If possible, also mention theories and principles used for designing the site. Keep in mind the needs of systematic reviewers and indexers by including important synonyms. (Note: Only report in the abstract what the main paper is reporting. If this information is missing from the main body of text, consider adding it)

	1	2	3	4	5	
subitem not at all important	○	○	○	○	○	essential

Does your paper address subitem 1b-i? *

Copy and paste relevant sections from the manuscript abstract (include quotes in quotation marks "like this" to indicate direct quotes from your manuscript), or elaborate on this item by providing additional information not in the ms, or briefly explain why the item is not applicable/relevant for your study

Participants received neurofeedback-assisted online mindfulness intervention, conventional online mindfulness interventions, or general mental health education over the next six weeks. A self-reported questionnaire and electroencephalogram (EEG) were used to evaluate the mental health status at baseline, immediately after intervention, 1 month and 3 months after intervention.

1b-ii) Level of human involvement in the METHODS section of the ABSTRACT

Clarify the level of human involvement in the abstract, e.g., use phrases like “fully automated” vs. “therapist/nurse/care provider/physician-assisted” (mention number and expertise of providers involved, if any). (Note: Only report in the abstract what the main paper is reporting. If this information is missing from the main body of text, consider adding it)

	1	2	3	4	5	
subitem not at all important	○	○	○	○	○	essential

Does your paper address subitem 1b-ii?

Copy and paste relevant sections from the manuscript abstract (include quotes in quotation marks "like this" to indicate direct quotes from your manuscript), or elaborate on this item by providing additional information not in the ms, or briefly explain why the item is not applicable/relevant for your study

您的回答

1b-iii) Open vs. closed, web-based (self-assessment) vs. face-to-face assessments in the METHODS section of the ABSTRACT

Mention how participants were recruited (online vs. offline), e.g., from an open access website or from a clinic or a closed online user group (closed usergroup trial), and clarify if this was a purely web-based trial, or there were face-to-face components (as part of the intervention or for assessment). Clearly say if outcomes were self-assessed through questionnaires (as common in web-based trials). Note: In traditional offline trials, an open trial (open-label trial) is a type of clinical trial in which both the researchers and participants know which treatment is being administered. To avoid confusion, use “blinded” or “unblinded” to indicated the level of blinding instead of “open”, as “open” in web-based trials usually refers to “open access” (i.e. participants can self-enrol). (Note: Only report in the abstract what the main paper is reporting. If this information is missing from the main body of text, consider adding it)

	1	2	3	4	5	
subitem not at all important	○	○	○	○	○	essential

Does your paper address subitem 1b-iii?

Copy and paste relevant sections from the manuscript abstract (include quotes in quotation marks "like this" to indicate direct quotes from your manuscript), or elaborate on this item by providing additional information not in the ms, or briefly explain why the item is not applicable/relevant for your study

您的回答

1b-iv) RESULTS section in abstract must contain use data

Report number of participants enrolled/assessed in each group, the use/uptake of the intervention (e.g., attrition/adherence metrics, use over time, number of logins etc.), in addition to primary/secondary outcomes. (Note: Only report in the abstract what the main paper is reporting. If this information is missing from the main body of text, consider adding it)

	1	2	3	4	5	
subitem not at all important	○	○	○	○	○	essential

Does your paper address subitem 1b-iv?

Copy and paste relevant sections from the manuscript abstract (include quotes in quotation marks "like this" to indicate direct quotes from your manuscript), or elaborate on this item by providing additional information not in the ms, or briefly explain why the item is not applicable/relevant for your study

您的回答

1b-v) CONCLUSIONS/DISCUSSION in abstract for negative trials

Conclusions/Discussions in abstract for negative trials: Discuss the primary outcome - if the trial is negative (primary outcome not changed), and the intervention was not used, discuss whether negative results are attributable to lack of uptake and discuss reasons. (Note: Only report in the abstract what the main paper is reporting. If this information is missing from the main body of text, consider adding it)

	1	2	3	4	5	
subitem not at all important	○	○	○	○	○	essential

Does your paper address subitem 1b-v?

Copy and paste relevant sections from the manuscript abstract (include quotes in quotation marks "like this" to indicate direct quotes from your manuscript), or elaborate on this item by providing additional information not in the ms, or briefly explain why the item is not applicable/relevant for your study

您的回答

INTRODUCTION

2a) In INTRODUCTION: Scientific background and explanation of rationale

2a-i) Problem and the type of system/solution

Describe the problem and the type of system/solution that is object of the study: intended as stand-alone intervention vs. incorporated in broader health care program? Intended for a particular patient population? Goals of the intervention, e.g., being more cost-effective to other interventions, replace or complement other solutions? (Note: Details about the intervention are provided in “Methods” under 5)

	1	2	3	4	5	
subitem not at all important	○	○	○	○	○	essential

Does your paper address subitem 2a-i? *

Copy and paste relevant sections from the manuscript (include quotes in quotation marks "like this" to indicate direct quotes from your manuscript), or elaborate on this item by providing additional information not in the ms, or briefly explain why the item is not applicable/relevant for your study

Mindfulness intervention is an emerging strategy that has a positive effect on reducing psychological distress and improving the mental health of nursing students. Mindfulness is defined as purposeful and conscious attention to everything at the moment, without making any judgments or reactions. It helps individuals become aware of both internal sensations, thoughts and feelings, as well as their external surroundings, allowing them to keep calm, reduce stress, and respond with conscious choice rather than automatic reaction. Mindfulness interventions, including meditation, yoga, and other forms, have been universally acknowledged as effective protective approaches for promoting mental health across various populations. Studies revealed that mindfulness interventions can effectively reduce stress, depression, and anxiety symptoms among nursing students. A meta-analysis conducted with 1,579 studies showed that mindfulness interventions comprised the largest sample sizes, and displayed the highest level of evidence, compared to other interventions that aimed at alleviating the mental health problems of nursing students. Additionally, medical students favored online mental health interventions than face-to-face mental consultations for mental health support owing to fears of stigmatization, shame, barriers of time and place, and distrust of intervention offers. Online mindfulness interventions could overcome some of the above barriers as they can be accessed anonymously from anywhere and at any time, and can be used individually in a safe setting. Therefore, online mindfulness intervention is an ideal and promising approach to promote mental health and alleviate the mental problems of nursing students.

2a-ii) Scientific background, rationale: What is known about the (type of) system

Scientific background, rationale: What is known about the (type of) system that is the object of the study (be sure to discuss the use of similar systems for other conditions/diagnoses, if appropiate), motivation for the study, i.e. what are the reasons for and what is the context for this specific study, from which stakeholder viewpoint is the study performed, potential impact of findings [2]. Briefly justify the choice of the comparator.

	1	2	3	4	5	
subitem not at all important	○	○	○	○	○	essential

Does your paper address subitem 2a-ii? *

Copy and paste relevant sections from the manuscript (include quotes in quotation marks "like this" to indicate direct quotes from your manuscript), or elaborate on this item by providing additional information not in the ms, or briefly explain why the item is not applicable/relevant for your study

Nursing students showed significantly higher rates of mental health problems compared to medical students in other majors, as they are faced with more serious mental health challenges due to social prejudice from their families and peers regarding their future profession. According to a meta-analysis of 13,247 nursing students, the prevalence of depressive symptoms among them was 52%, far higher than that of the entire medical student population and the global historical data. In China, a cross-sectional study revealed that the prevalence of depressive and anxiety symptoms among Chinese nursing students in 2020 were 56.4% and 55.0%, highlighting the severity of this issue.
Identifying effective intervention methods for mental health problems in nursing students shall provide valuable insights for health educators, managers, and policymakers on how to prevent the occurrence of mental health problems in nursing students through daily interventions, and also provide timely and effective measures once at-risk students are identified.

2b) In INTRODUCTION: Specific objectives or hypotheses

Does your paper address CONSORT subitem 2b? *

Copy and paste relevant sections from the manuscript (include quotes in quotation marks "like this" to indicate direct quotes from your manuscript), or elaborate on this item by providing additional information not in the ms, or briefly explain why the item is not applicable/relevant for your study

our study aims to examine the short- and long-term effectiveness of the neurofeedback-assisted online mindfulness intervention (NAOM) and conventional online mindfulness intervention (COM) in reducing mental health problems and related physical symptoms among nursing students in China. We hypothesize that the six-week online mindfulness intervention, both the conventional and the neurofeedback-assisted could effectively improve mental health of nursing students in both the short term (immediately after the intervention) and long term (1 or 3 months after the intervention), and the neurofeedback technology could further improve the effectiveness of the online mindfulness intervention.

METHODS

3a) Description of trial design (such as parallel, factorial) including allocation ratio

Does your paper address CONSORT subitem 3a? *

Copy and paste relevant sections from the manuscript (include quotes in quotation marks "like this" to indicate direct quotes from your manuscript), or elaborate on this item by providing additional information not in the ms, or briefly explain why the item is not applicable/relevant for your study

A three-armed, partially blind parallel-group RCT was conducted to compare the effectiveness of the NAOM, the COM, and the general mental health education in improving the mental health status of nursing students. A total of 172 nursing students completed the recruitment questionnaire (the baseline survey), of which 155 voluntarily participated in the subsequent intervention, with a 1:1:1 distribution across 3 groups: the NAOM group, the COM group and the control group.

3b) Important changes to methods after trial commencement (such as eligibility criteria), with reasons

Does your paper address CONSORT subitem 3b? *

Copy and paste relevant sections from the manuscript (include quotes in quotation marks "like this" to indicate direct quotes from your manuscript), or elaborate on this item by providing additional information not in the ms, or briefly explain why the item is not applicable/relevant for your study

There is no change to methods after trial commencement.

3b-i) Bug fixes, Downtimes, Content Changes

Bug fixes, Downtimes, Content Changes: ehealth systems are often dynamic systems. A description of changes to methods therefore also includes important changes made on the intervention or comparator during the trial (e.g., major bug fixes or changes in the functionality or content) (5-iii) and other “unexpected events” that may have influenced study design such as staff changes, system failures/downtimes, etc. [2].

	1	2	3	4	5	
subitem not at all important	○	○	○	○	○	essential

Does your paper address subitem 3b-i?

Copy and paste relevant sections from the manuscript (include quotes in quotation marks "like this" to indicate direct quotes from your manuscript), or elaborate on this item by providing additional information not in the ms, or briefly explain why the item is not applicable/relevant for your study

您的回答

4a) Eligibility criteria for participants

Does your paper address CONSORT subitem 4a? *

Copy and paste relevant sections from the manuscript (include quotes in quotation marks "like this" to indicate direct quotes from your manuscript), or elaborate on this item by providing additional information not in the ms, or briefly explain why the item is not applicable/relevant for your study

The eligibility criteria were as follows: (1) Full-time nursing students over 18 years old; (2) Ability to independently cooperate to complete various questionnaire assessments and EEG collection; (3) Possession of a mobile phone with functional Bluetooth and connection to the internet at any time; (4) Right-handedness (considering that the brain structures and EEG among left- and right- handedness are different); (5) No mindfulness-related intervention or training received within 6 months before the enrollment in this study; and (5)Willingness to participate throughout the entire process, providing informed consent, and signing the electronic version of the informed consent form as required by the ethics committee. Those who were in other health promotions or clinical intervention studies, suspended or dropped out of school during the research period, or had obvious scars on the forehead or other conditions unsuitable for EEG collection were excluded.

4a-i) Computer / Internet literacy

Computer / Internet literacy is often an implicit “de facto” eligibility criterion - this should be explicitly clarified.

	1	2	3	4	5	
subitem not at all important	○	○	○	○	○	essential

Does your paper address subitem 4a-i?

Copy and paste relevant sections from the manuscript (include quotes in quotation marks "like this" to indicate direct quotes from your manuscript), or elaborate on this item by providing additional information not in the ms, or briefly explain why the item is not applicable/relevant for your study

您的回答

4a-ii) Open vs. closed, web-based vs. face-to-face assessments:

Open vs. closed, web-based vs. face-to-face assessments: Mention how participants were recruited (online vs. offline), e.g., from an open access website or from a clinic, and clarify if this was a purely web-based trial, or there were face-to-face components (as part of the intervention or for assessment), i.e., to what degree got the study team to know the participant. In online-only trials, clarify if participants were quasi-anonymous and whether having multiple identities was possible or whether technical or logistical measures (e.g., cookies, email confirmation, phone calls) were used to detect/prevent these.

	1	2	3	4	5	
subitem not at all important	○	○	○	○	○	essential

Does your paper address subitem 4a-ii? *

Copy and paste relevant sections from the manuscript (include quotes in quotation marks "like this" to indicate direct quotes from your manuscript), or elaborate on this item by providing additional information not in the ms, or briefly explain why the item is not applicable/relevant for your study

Participants were recruited from a faculty of nursing at a medical college in Beijing, China by using WeChat-based and offline publicity from March 1 to 16, 2024. Researchers distributed digital posters through WeChat groups and also put up print posters in student dormitories, lecture halls, and classrooms to provide information regarding this study for nursing students. Interested students can scan the QR code on the posters to fill out a recruitment questionnaire (baseline survey), which collected the demographic data, baseline mental health status, and informed consent from the potential participants of the subsequent intervention. The entire recruitment process was automated to eliminate human contact and maintain scalability.

4a-iii) Information giving during recruitment

Information given during recruitment. Specify how participants were briefed for recruitment and in the informed consent procedures (e.g., publish the informed consent documentation as appendix, see also item X26), as this information may have an effect on user self-selection, user expectation and may also bias results.

	1	2	3	4	5	
subitem not at all important	○	○	○	○	○	essential

Does your paper address subitem 4a-iii?

Copy and paste relevant sections from the manuscript (include quotes in quotation marks "like this" to indicate direct quotes from your manuscript), or elaborate on this item by providing additional information not in the ms, or briefly explain why the item is not applicable/relevant for your study

您的回答

4b) Settings and locations where the data were collected

Does your paper address CONSORT subitem 4b? *

Copy and paste relevant sections from the manuscript (include quotes in quotation marks "like this" to indicate direct quotes from your manuscript), or elaborate on this item by providing additional information not in the ms, or briefly explain why the item is not applicable/relevant for your study

Assessments were administered three times: at immediately post-intervention, 1 month and 3 months post-intervention. All participants received automatic messages with a follow-up questionnaire link, and those who did not complete the questionnaire within 3 days would also continuously receive reminder messages sent by the PUMCQS applet.

4b-i) Report if outcomes were (self-)assessed through online questionnaires

Clearly report if outcomes were (self-)assessed through online questionnaires (as common in web-based trials) or otherwise.

	1	2	3	4	5	
subitem not at all important	○	○	○	○	○	essential

Does your paper address subitem 4b-i? *

Copy and paste relevant sections from the manuscript (include quotes in quotation marks "like this" to indicate direct quotes from your manuscript), or elaborate on this item by providing additional information not in the ms, or briefly explain why the item is not applicable/relevant for your study

Meanwhile, demographic information, including age, sex, ethnicity, degree, student cadre status, only-child status, family annual income, situation of smoking and alcohol use, reason for choosing a nursing major, etc., was obtained using self-reported questionnaires.

4b-ii) Report how institutional affiliations are displayed

Report how institutional affiliations are displayed to potential participants [on ehealth media], as affiliations with prestigious hospitals or universities may affect volunteer rates, use, and reactions with regards to an intervention.(Not a required item – describe only if this may bias results)

	1	2	3	4	5	
subitem not at all important	○	○	○	○	○	essential

Does your paper address subitem 4b-ii?

Copy and paste relevant sections from the manuscript (include quotes in quotation marks "like this" to indicate direct quotes from your manuscript), or elaborate on this item by providing additional information not in the ms, or briefly explain why the item is not applicable/relevant for your study

您的回答

5) The interventions for each group with sufficient details to allow replication, including how and when they were actually administered

5-i) Mention names, credential, affiliations of the developers, sponsors, and owners

Mention names, credential, affiliations of the developers, sponsors, and owners [6] (if authors/evaluators are owners or developer of the software, this needs to be declared in a “Conflict of interest” section or mentioned elsewhere in the manuscript).

	1	2	3	4	5	
subitem not at all important	○	○	○	○	○	essential

Does your paper address subitem 5-i?

Copy and paste relevant sections from the manuscript (include quotes in quotation marks "like this" to indicate direct quotes from your manuscript), or elaborate on this item by providing additional information not in the ms, or briefly explain why the item is not applicable/relevant for your study

您的回答

5-ii) Describe the history/development process

Describe the history/development process of the application and previous formative evaluations (e.g., focus groups, usability testing), as these will have an impact on adoption/use rates and help with interpreting results.

	1	2	3	4	5	
subitem not at all important	○	○	○	○	○	essential

Does your paper address subitem 5-ii?

Copy and paste relevant sections from the manuscript (include quotes in quotation marks "like this" to indicate direct quotes from your manuscript), or elaborate on this item by providing additional information not in the ms, or briefly explain why the item is not applicable/relevant for your study

您的回答

5-iii) Revisions and updating

Revisions and updating. Clearly mention the date and/or version number of the application/intervention (and comparator, if applicable) evaluated, or describe whether the intervention underwent major changes during the evaluation process, or whether the development and/or content was "frozen" during the trial. Describe dynamic components such as news feeds or changing content which may have an impact on the replicability of the intervention (for unexpected events see item 3b).

	1	2	3	4	5	
subitem not at all important	○	○	○	○	○	essential

Does your paper address subitem 5-iii?

Copy and paste relevant sections from the manuscript (include quotes in quotation marks "like this" to indicate direct quotes from your manuscript), or elaborate on this item by providing additional information not in the ms, or briefly explain why the item is not applicable/relevant for your study

您的回答

5-iv) Quality assurance methods

Provide information on quality assurance methods to ensure accuracy and quality of information provided [1], if applicable.

	1	2	3	4	5	
subitem not at all important	○	○	○	○	○	essential

Does your paper address subitem 5-iv?

Copy and paste relevant sections from the manuscript (include quotes in quotation marks "like this" to indicate direct quotes from your manuscript), or elaborate on this item by providing additional information not in the ms, or briefly explain why the item is not applicable/relevant for your study

您的回答

5-v) Ensure replicability by publishing the source code, and/or providing screenshots/screen-capture video, and/or providing flowcharts of the algorithms used

Ensure replicability by publishing the source code, and/or providing screenshots/screen-capture video, and/or providing flowcharts of the algorithms used. Replicability (i.e., other researchers should in principle be able to replicate the study) is a hallmark of scientific reporting.

	1	2	3	4	5	
subitem not at all important	○	○	○	○	○	essential

Does your paper address subitem 5-v?

Copy and paste relevant sections from the manuscript (include quotes in quotation marks "like this" to indicate direct quotes from your manuscript), or elaborate on this item by providing additional information not in the ms, or briefly explain why the item is not applicable/relevant for your study

您的回答

5-vi) Digital preservation

Digital preservation: Provide the URL of the application, but as the intervention is likely to change or disappear over the course of the years; also make sure the intervention is archived (Internet Archive, webcitation.org, and/or publishing the source code or screenshots/videos alongside the article). As pages behind login screens cannot be archived, consider creating demo pages which are accessible without login.

	1	2	3	4	5	
subitem not at all important	○	○	○	○	○	essential

Does your paper address subitem 5-vi?

Copy and paste relevant sections from the manuscript (include quotes in quotation marks "like this" to indicate direct quotes from your manuscript), or elaborate on this item by providing additional information not in the ms, or briefly explain why the item is not applicable/relevant for your study

您的回答

5-vii) Access

Access: Describe how participants accessed the application, in what setting/context, if they had to pay (or were paid) or not, whether they had to be a member of specific group. If known, describe how participants obtained “access to the platform and Internet” [1]. To ensure access for editors/reviewers/readers, consider to provide a “backdoor” login account or demo mode for reviewers/readers to explore the application (also important for archiving purposes, see vi).

	1	2	3	4	5	
subitem not at all important	○	○	○	○	○	essential

Does your paper address subitem 5-vii? *

Copy and paste relevant sections from the manuscript (include quotes in quotation marks "like this" to indicate direct quotes from your manuscript), or elaborate on this item by providing additional information not in the ms, or briefly explain why the item is not applicable/relevant for your study

Once a potential participant met the inclusion and exclusion criteria, provided informed consent, and finished the baseline questionnaire and EEG acquisition, the individual was recruited and scheduled in the study. Researchers then enter the participant's personal information into the "PUMC Questionnaire Survey" (PUMCQS) applet and conduct randomization, in which he/she was randomized to one of the three groups. Participants in the NAOM group, and the COM group received 6-week online mindfulness intervention, and those in the control group were provided during the same period of time with general mental health education materials sent by the PUMCQS applet.

5-viii) Mode of delivery, features/functionalities/components of the intervention and comparator, and the theoretical framework

Describe mode of delivery, features/functionalities/components of the intervention and comparator, and the theoretical framework [6] used to design them (instructional strategy [1], behaviour change techniques, persuasive features, etc., see e.g., [7, 8] for terminology). This includes an in-depth description of the content (including where it is coming from and who developed it) [1]," whether [and how] it is tailored to individual circumstances and allows users to track their progress and receive feedback" [6]. This also includes a description of communication delivery channels and – if computer-mediated communication is a component – whether communication was synchronous or asynchronous [6]. It also includes information on presentation strategies [1], including page design principles, average amount of text on pages, presence of hyperlinks to other resources, etc. [1].

	1	2	3	4	5	
subitem not at all important	○	○	○	○	○	essential

Does your paper address subitem 5-viii? *

Copy and paste relevant sections from the manuscript (include quotes in quotation marks "like this" to indicate direct quotes from your manuscript), or elaborate on this item by providing additional information not in the ms, or briefly explain why the item is not applicable/relevant for your study

Development of a six-week online mindfulness intervention program
Experienced epidemiologists and psychologists jointly discussed and designed a six-week online mindfulness intervention program for this study. It consisted of 6 sessions of online lessons (one session per week, and two lessons per session), which focused on four key aspects of mindfulness intervention: attention control, self-awareness, acceptance and discernment, and emotion regulation. The online lessons were given through 12 instructional videos and 9 audios on theoretical knowledge, meditation practice, mindful eating, mindful stretching, and mindful yoga, which a psychiatrist, certified in the Training of Mindfulness Facilitation program at the Mindful Awareness Research Center of University of California, Los Angeles (UCLA), were invited to record for mindfulness practice. This program has obtained a work registration certificate from the National Copyright Administration of the People's Republic of China (registration number: GZDZ-2021-I-00193781). See Table 1 for a description of this course.

5-ix) Describe use parameters

Describe use parameters (e.g., intended "doses" and optimal timing for use). Clarify what instructions or recommendations were given to the user, e.g., regarding timing, frequency, heaviness of use, if any, or was the intervention used ad libitum.

	1	2	3	4	5	
subitem not at all important	○	○	○	○	○	essential

Does your paper address subitem 5-ix?

Copy and paste relevant sections from the manuscript (include quotes in quotation marks "like this" to indicate direct quotes from your manuscript), or elaborate on this item by providing additional information not in the ms, or briefly explain why the item is not applicable/relevant for your study

您的回答

5-x) Clarify the level of human involvement

Clarify the level of human involvement (care providers or health professionals, also technical assistance) in the e-intervention or as co-intervention (detail number and expertise of professionals involved, if any, as well as "type of assistance offered, the timing and frequency of the support, how it is initiated, and the medium by which the assistance is delivered". It may be necessary to distinguish between the level of human involvement required for the trial, and the level of human involvement required for a routine application outside of a RCT setting (discuss under item 21 – generalizability).

	1	2	3	4	5	
subitem not at all important	○	○	○	○	○	essential

Does your paper address subitem 5-x?

Copy and paste relevant sections from the manuscript (include quotes in quotation marks "like this" to indicate direct quotes from your manuscript), or elaborate on this item by providing additional information not in the ms, or briefly explain why the item is not applicable/relevant for your study

您的回答

5-xi) Report any prompts/reminders used

Report any prompts/reminders used: Clarify if there were prompts (letters, emails, phone calls, SMS) to use the application, what triggered them, frequency etc. It may be necessary to distinguish between the level of prompts/reminders required for the trial, and the level of prompts/reminders for a routine application outside of a RCT setting (discuss under item 21 – generalizability).

	1	2	3	4	5	
subitem not at all important	○	○	○	○	○	essential

Does your paper address subitem 5-xi? *

Copy and paste relevant sections from the manuscript (include quotes in quotation marks "like this" to indicate direct quotes from your manuscript), or elaborate on this item by providing additional information not in the ms, or briefly explain why the item is not applicable/relevant for your study

Participants in the NAOM group, and the COM group received 6-week online mindfulness intervention, and those in the control group were provided during the same period of time with general mental health education materials sent by the PUMCQS applet. This applet console could track the viewing and listening time of each participant, and those who did not finish the learning content within 3 days after the deadline would receive automatic reminder messages from the PUMCQS applet.

5-xii) Describe any co-interventions (incl. training/support)

Describe any co-interventions (incl. training/support): Clearly state any interventions that are provided in addition to the targeted eHealth intervention, as ehealth intervention may not be designed as stand-alone intervention. This includes training sessions and support [1]. It may be necessary to distinguish between the level of training required for the trial, and the level of training for a routine application outside of a RCT setting (discuss under item 21 – generalizability.

	1	2	3	4	5	
subitem not at all important	○	○	○	○	○	essential

Does your paper address subitem 5-xii? *

Copy and paste relevant sections from the manuscript (include quotes in quotation marks "like this" to indicate direct quotes from your manuscript), or elaborate on this item by providing additional information not in the ms, or briefly explain why the item is not applicable/relevant for your study

Neurofeedback-assisted online mindfulness intervention (NAOM)
Participants in the NAOM group were required to participate in the same 6-week online mindfulness program. Additionally, they were asked to wear a FocusZen headband developed by Zhejiang Qiangnao Technology Co., Ltd when practicing 6 mediation audios. The device continuously collected the EEG signals of the participants through a logged-in mobile app named “OxyZen”. The headband and the mobile app used in this study are shown in Figures 2 and 3.
The FocusZen headband contains 3 electrodes which are positioned on the prefrontal cortex (Fp1, Fp2, Fpz sites, 10-20 system), and EEG signals can be monitored at a sampling rate of 250Hz. Ocular, muscular, and other types of artifacts can be detected by an automatic removal algorithm in this device. The digitized EEG data is processed on the host device to generate a “mindfulness index” ranging from 0 to 100, calculated using a Convolutional Neural Network and proprietary Artificial Intelligence models embedded in the “OxyZen” app. These models were developed through iterative analysis of extensive EEG data from mindfulness practitioners and experts at various skill levels, across diverse cognitive tasks. A total of 6 mindfulness meditation audios were conducted using the “OxyZen” app, during which the participants were required to wear the FocusZen headband, and collect their EEG data during meditation exercises. Upon completing each meditation practice audio, participants obtained a neurofeedback report with curve plots that visually display their mindfulness index and multidimensional brain activity analyses, providing an objective assessment of their mindfulness practice status and actionable insights to help optimize their practice for future sessions. All participants in this group received a training on the use of the FocusZen headband and the “OxyZen” app before the intervention. For the remaining videos and audios that did not involve meditation practice, participants in the NAOM group only needed to complete the online program through the PUMCQS applet like the COM group.

6a) Completely defined pre-specified primary and secondary outcome measures, including how and when they were assessed

Does your paper address CONSORT subitem 6a? *

Copy and paste relevant sections from the manuscript (include quotes in quotation marks "like this" to indicate direct quotes from your manuscript), or elaborate on this item by providing additional information not in the ms, or briefly explain why the item is not applicable/relevant for your study

Assessments were administered three times: at immediately post-intervention, 1 month and 3 months post-intervention. All participants received automatic messages with a follow-up questionnaire link, and those who did not complete the questionnaire within 3 days would also continuously receive reminder messages sent by the PUMCQS applet.

Outcomes

Primary outcomes

Depressive symptoms

Depressive symptoms were assessed by the 9-item Patient Health Questionnaire (PHQ-9), which was developed by Kroenke in 2001 and has been validated among various Chinese populations (34-36). The items of the PHQ-9 are scored on a 4-point Likert scale with a range of 0 to 3. Higher total scores indicated higher levels of depressive symptoms. Cronbach's α of the instrument was 0.881, 0.860, 0.906, and 0.912, respectively of the 4 assessments.

Anxiety symptoms

The Generalized Anxiety Disorder Questionnaire (GAD-7), comprising 7 items and measured on a 4-point Likert Scale from 0-3, was used to evaluate the anxiety symptoms during the past two weeks of each participant. The scores of the instrument range from 0 to 21, and higher total scores indicated higher levels of anxiety(37). This instrument has been demonstrated to be reliable and valid among the Chinese population(38, 39). In this study, the Cronbach's α of this instrument was 0.931, 0.892, 0.946, and 0.937, respectively of the 4 assessments.

Secondary outcomes

Fatigue

The Fatigue Scale-14 (FS-14) has been proven valid and reliable among the general population in China (40), which consists of 14 items, with each item rated on a 2-point scale (0=No, 1=Yes). This scale is most commonly used and studied for the evaluation of fatigue severity during the past two weeks(41). In this study, the sum of scores on each item was calculated and higher total scores reflect higher levels of fatigue. The Cronbach's α of the instrument was 0.802, 0.824, 0.868, and 0.857, respectively of the 4 assessments.

Personality-Neuroticism

Personality-Neuroticism was assessed by the NEO Five-Factor Inventory (NEO-FFI), which was developed by Costa and McCrae in 1987 and adapted for the Chinese population by Jianxin Zhang(42). This instrument is sensitive to personality across neuroticism and higher total scores indicate that an individual is more likely and easily to feel nervous and anxious. The Cronbach's α of the instrument was 0.801, 0.868, 0.884, and 0.887, respectively of the 4 assessments.

Quality of sleep

Designed by Li, et al., the Self-Rating Scale of Sleep (SRSS) was adapted to evaluate the quality of sleep among students in the past month. This instrument contains 10 items with each item rated on a 5-point Likert scales ranging from 1 to 5, and higher total scores of SRSS indicate worse quality of sleep(43). The Cronbach's α of the instrument in this study was 0.660, 0.816, 0.794, and 0.795, respectively of the 4 assessments.

Resilience

The 10-item Connor-Davidson Resilience Scale (CD-RISC-10) was used to measure participants' psychological resilience, which was developed by Connor, et al. in 2003 with 25 items and was reduced to 10 items by Campbell-Sills, et al. in 2007(44, 45). This instrument contains 10 items which are 4-point Likert scales ranging from 1 to 4 and has been validated among parents of children with cancer diagnosis and earthquake victims in China (46, 47). Higher total scores of CD-RISC-10 indicate better psychological resilience. The Cronbach's α of the instrument in this study was 0.924, 0.944, 0.957, and 0.963, respectively of the 4 assessments.

Mindfulness

The 5-item Mindful Awareness Attention Scale (MAAS-5) was developed to evaluate the mindfulness level by Brown, et al. in 2003 and was simplified to 5 items by Tomás Caycho-Rodríguez, et al. in 2021(48, 49). This instrument was translated into Chinese through a rigorous process and adapted in Chinese nursing students by our research team(50). MAAS-5 contains 5 items and each item was rated on a 7-point Likert scale (0=never to 6=always), with higher scores reflecting a lower level of mindfulness. The Cronbach's α of the instrument in this study was 0.829, 0.855, 0.898, and 0.893, respectively of the 4 times. Meanwhile, demographic information, including age, sex, ethnicity, degree, student cadre status, only-child status, family annual income, situation of smoking and alcohol use, reason for choosing a nursing major, etc., was obtained using self-reported questionnaires. The acceptance of the neurofeedback-assisted online mindfulness intervention using headband and the "OxyZen" app of the NAOM group was assessed by using the theory of TAM from 4 dimensions: perceived ease of use, perceived usefulness, attitude, and behavioral intention(51). Additionally, EEG data of resting state with eyes closed and during meditation state of all participants was also recorded simultaneously in this study. The power spectra of theta (4-8 Hz), alpha (8-13 Hz), beta (13-30 Hz), and gamma (>30 Hz) frequency bands were also calculated.

6a-i) Online questionnaires: describe if they were validated for online use and apply CHERRIES items to describe how the questionnaires were designed/deployed

If outcomes were obtained through online questionnaires, describe if they were validated for online use and apply CHERRIES items to describe how the questionnaires were designed/deployed [9].

	1	2	3	4	5	
subitem not at all important	○	○	○	○	○	essential

Does your paper address subitem 6a-i?

Copy and paste relevant sections from manuscript text

您的回答

6a-ii) Describe whether and how “use” (including intensity of use/dosage) was defined/measured/monitored

Describe whether and how “use” (including intensity of use/dosage) was defined/measured/monitored (logins, logfile analysis, etc.). Use/adoption metrics are important process outcomes that should be reported in any ehealth trial.

	1	2	3	4	5	
subitem not at all important	○	○	○	○	○	essential

Does your paper address subitem 6a-ii?

Copy and paste relevant sections from manuscript text

您的回答

6a-iii) Describe whether, how, and when qualitative feedback from participants was obtained

Describe whether, how, and when qualitative feedback from participants was obtained (e.g., through emails, feedback forms, interviews, focus groups).

	1	2	3	4	5	
subitem not at all important	○	○	○	○	○	essential

Does your paper address subitem 6a-iii?

Copy and paste relevant sections from manuscript text

您的回答

6b) Any changes to trial outcomes after the trial commenced, with reasons

Does your paper address CONSORT subitem 6b? *

Copy and paste relevant sections from the manuscript (include quotes in quotation marks "like this" to indicate direct quotes from your manuscript), or elaborate on this item by providing additional information not in the ms, or briefly explain why the item is not applicable/relevant for your study

There is no change to trial outcomes after the trial commenced.

7a) How sample size was determined

NPT: When applicable, details of whether and how the clustering by care provides or centers was addressed

7a-i) Describe whether and how expected attrition was taken into account when calculating the sample size

Describe whether and how expected attrition was taken into account when calculating the sample size.

	1	2	3	4	5	
subitem not at all important	○	○	○	○	○	essential

Does your paper address subitem 7a-i?

Copy and paste relevant sections from manuscript title (include quotes in quotation marks "like this" to indicate direct quotes from your manuscript), or elaborate on this item by providing additional information not in the ms, or briefly explain why the item is not applicable/relevant for your study

您的回答

7b) When applicable, explanation of any interim analyses and stopping guidelines

Does your paper address CONSORT subitem 7b? *

Copy and paste relevant sections from the manuscript (include quotes in quotation marks "like this" to indicate direct quotes from your manuscript), or elaborate on this item by providing additional information not in the ms, or briefly explain why the item is not applicable/relevant for your study

Assessments were administered three times: at immediately post-intervention, 1 month and 3 months post-intervention. All participants received automatic messages with a follow-up questionnaire link, and those who did not complete the questionnaire within 3 days would also continuously receive reminder messages sent by the PUMCQS applet.

Intention-to-treat principles were applied to determine the research samples of each group.

8a) Method used to generate the random allocation sequence

NPT: When applicable, how care providers were allocated to each trial group

Does your paper address CONSORT subitem 8a? *

Copy and paste relevant sections from the manuscript (include quotes in quotation marks "like this" to indicate direct quotes from your manuscript), or elaborate on this item by providing additional information not in the ms, or briefly explain why the item is not applicable/relevant for your study

Randomization and allocation were automated and carried out independently. All participants were randomized (1:1:1 allocation ratio) using a blocked randomization sequence at the PUMCQS applet, and the trial coordinators of this study enrolled the participants after the randomization.

8b) Type of randomisation; details of any restriction (such as blocking and block size)

Does your paper address CONSORT subitem 8b? *

Copy and paste relevant sections from the manuscript (include quotes in quotation marks "like this" to indicate direct quotes from your manuscript), or elaborate on this item by providing additional information not in the ms, or briefly explain why the item is not applicable/relevant for your study

Randomization and allocation were automated and carried out independently. All participants were randomized (1:1:1 allocation ratio) using a blocked randomization sequence at the PUMCQS applet, and the trial coordinators of this study enrolled the participants after the randomization.

9) Mechanism used to implement the random allocation sequence (such as sequentially numbered containers), describing any steps taken to conceal the sequence until interventions were assigned

Does your paper address CONSORT subitem 9? *

Copy and paste relevant sections from the manuscript (include quotes in quotation marks "like this" to indicate direct quotes from your manuscript), or elaborate on this item by providing additional information not in the ms, or briefly explain why the item is not applicable/relevant for your study

Randomization and blinding
Randomization and allocation were automated and carried out independently. All participants were randomized (1:1:1 allocation ratio) using a blocked randomization sequence at the PUMCQS applet, and the trial coordinators of this study enrolled the participants after the randomization. Participants and the trial coordinators were not blind to the group allocation due to the intrinsic limitations in intervention methods. All other members of the research team including data analysts were blind to the group allocation during the intervention and the follow-ups. The groups were labeled as “A”, “B” and “C” during the statistical analysis.

10) Who generated the random allocation sequence, who enrolled participants, and who assigned participants to interventions

Does your paper address CONSORT subitem 10? *

Copy and paste relevant sections from the manuscript (include quotes in quotation marks "like this" to indicate direct quotes from your manuscript), or elaborate on this item by providing additional information not in the ms, or briefly explain why the item is not applicable/relevant for your study

Randomization and blinding
Randomization and allocation were automated and carried out independently. All participants were randomized (1:1:1 allocation ratio) using a blocked randomization sequence at the PUMCQS applet, and the trial coordinators of this study enrolled the participants after the randomization. Participants and the trial coordinators were not blind to the group allocation due to the intrinsic limitations in intervention methods. All other members of the research team including data analysts were blind to the group allocation during the intervention and the follow-ups. The groups were labeled as “A”, “B” and “C” during the statistical analysis.

11a) If done, who was blinded after assignment to interventions (for example, participants, care providers, those assessing outcomes) and how

NPT: Whether or not administering co-interventions were blinded to group assignment

11a-i) Specify who was blinded, and who wasn’t

Specify who was blinded, and who wasn’t. Usually, in web-based trials it is not possible to blind the participants [1, 3] (this should be clearly acknowledged), but it may be possible to blind outcome assessors, those doing data analysis or those administering co-interventions (if any).

	1	2	3	4	5	
subitem not at all important	○	○	○	○	○	essential

Does your paper address subitem 11a-i? *

Copy and paste relevant sections from the manuscript (include quotes in quotation marks "like this" to indicate direct quotes from your manuscript), or elaborate on this item by providing additional information not in the ms, or briefly explain why the item is not applicable/relevant for your study

. Participants and the trial coordinators were not blind to the group allocation due to the intrinsic limitations in intervention methods. All other members of the research team including data analysts were blind to the group allocation during the intervention and the follow-ups. The groups were labeled as “A”, “B” and “C” during the statistical analysis.

11a-ii) Discuss e.g., whether participants knew which intervention was the “intervention of interest” and which one was the “comparator”

Informed consent procedures (4a-ii) can create biases and certain expectations - discuss e.g., whether participants knew which intervention was the “intervention of interest” and which one was the “comparator”.

	1	2	3	4	5	
subitem not at all important	○	○	○	○	○	essential

Does your paper address subitem 11a-ii?

Copy and paste relevant sections from the manuscript (include quotes in quotation marks "like this" to indicate direct quotes from your manuscript), or elaborate on this item by providing additional information not in the ms, or briefly explain why the item is not applicable/relevant for your study

您的回答

11b) If relevant, description of the similarity of interventions

(this item is usually not relevant for ehealth trials as it refers to similarity of a placebo or sham intervention to a active medication/intervention)

Does your paper address CONSORT subitem 11b? *

Copy and paste relevant sections from the manuscript (include quotes in quotation marks "like this" to indicate direct quotes from your manuscript), or elaborate on this item by providing additional information not in the ms, or briefly explain why the item is not applicable/relevant for your study

This item is not relevant.

12a) Statistical methods used to compare groups for primary and secondary outcomes

NPT: When applicable, details of whether and how the clustering by care providers or centers was addressed

Does your paper address CONSORT subitem 12a? *

Copy and paste relevant sections from the manuscript (include quotes in quotation marks "like this" to indicate direct quotes from your manuscript), or elaborate on this item by providing additional information not in the ms, or briefly explain why the item is not applicable/relevant for your study

Statistical analysis
Intention-to-treat principles were applied to determine the research samples of each group. Descriptive analyses were used to describe the demographic characteristics of the participants. One-way analyses of variance (ANOVA), Chi-square tests, and Fisher's exact tests were used to examine the comparability of the baseline demographic variables and psychological measurements among the 3 groups. The generalized estimating equation (GEE) was applied to examine the effects of interventions on all outcomes. In this study, GEE was used to evaluate the main effects of intervention and time, and their interaction effects. Simple effects with multiple comparisons using the least significant difference (LSD) method were used to evaluate the effect of intervention and time when the interaction effect was statistically significant. When the interaction was absent, the effect of the group and time were reassessed. Statistical significance was assessed by two-sided tests with a P-value <0.05. All statistical analyses were completed with IBM SPSS Statistics 24.0 (IBM Corp) and SAS 9.4.

12a-i) Imputation techniques to deal with attrition / missing values

Imputation techniques to deal with attrition / missing values: Not all participants will use the intervention/comparator as intended and attrition is typically high in ehealth trials. Specify how participants who did not use the application or dropped out from the trial were treated in the statistical analysis (a complete case analysis is strongly discouraged, and simple imputation techniques such as LOCF may also be problematic [4]).

	1	2	3	4	5	
subitem not at all important	○	○	○	○	○	essential

Does your paper address subitem 12a-i? *

Copy and paste relevant sections from the manuscript (include quotes in quotation marks "like this" to indicate direct quotes from your manuscript), or elaborate on this item by providing additional information not in the ms, or briefly explain why the item is not applicable/relevant for your study

Intention-to-treat principles were applied to determine the research samples of each group.

12b) Methods for additional analyses, such as subgroup analyses and adjusted analyses

Does your paper address CONSORT subitem 12b? *

Copy and paste relevant sections from the manuscript (include quotes in quotation marks "like this" to indicate direct quotes from your manuscript), or elaborate on this item by providing additional information not in the ms, or briefly explain why the item is not applicable/relevant for your study

The generalized estimating equation (GEE) was applied to examine the effects of interventions on all outcomes. In this study, GEE was used to evaluate the main effects of intervention and time, and their interaction effects. Simple effects with multiple comparisons using the least significant difference (LSD) method were used to evaluate the effect of intervention and time when the interaction effect was statistically significant. When the interaction was absent, the effect of the group and time were reassessed. Statistical significance was assessed by two-sided tests with a P-value <0.05.

X26) REB/IRB Approval and Ethical Considerations [recommended as subheading under "Methods"] (not a CONSORT item)

X26-i) Comment on ethics committee approval

	1	2	3	4	5	
subitem not at all important	○	○	○	○	○	essential

Does your paper address subitem X26-i?

Copy and paste relevant sections from the manuscript (include quotes in quotation marks "like this" to indicate direct quotes from your manuscript), or elaborate on this item by providing additional information not in the ms, or briefly explain why the item is not applicable/relevant for your study

您的回答

x26-ii) Outline informed consent procedures

Outline informed consent procedures e.g., if consent was obtained offline or online (how? Checkbox, etc.?), and what information was provided (see 4a-ii). See [6] for some items to be included in informed consent documents.

	1	2	3	4	5	
subitem not at all important	○	○	○	○	○	essential

Does your paper address subitem X26-ii?

Copy and paste relevant sections from the manuscript (include quotes in quotation marks "like this" to indicate direct quotes from your manuscript), or elaborate on this item by providing additional information not in the ms, or briefly explain why the item is not applicable/relevant for your study

您的回答

X26-iii) Safety and security procedures

Safety and security procedures, incl. privacy considerations, and any steps taken to reduce the likelihood or detection of harm (e.g., education and training, availability of a hotline)

	1	2	3	4	5	
subitem not at all important	○	○	○	○	○	essential

Does your paper address subitem X26-iii?

Copy and paste relevant sections from the manuscript (include quotes in quotation marks "like this" to indicate direct quotes from your manuscript), or elaborate on this item by providing additional information not in the ms, or briefly explain why the item is not applicable/relevant for your study

您的回答

RESULTS

13a) For each group, the numbers of participants who were randomly assigned, received intended treatment, and were analysed for the primary outcome

NPT: The number of care providers or centers performing the intervention in each group and the number of patients treated by each care provider in each center

Does your paper address CONSORT subitem 13a? *

Copy and paste relevant sections from the manuscript (include quotes in quotation marks "like this" to indicate direct quotes from your manuscript), or elaborate on this item by providing additional information not in the ms, or briefly explain why the item is not applicable/relevant for your study

Of the 172 applicants screened for this study, there are 17 ineligibles, either unwilling to participate in the intervention or failing to meet the inclusion criteria. A total of 155 participants were initially included and randomly allocated to 1 of the 3 groups: 52 in the NAOM group, 51 in the COM group, and 52 in the control group. After randomization and allocation, 1 participant in the NAOM group, 3 in the COM group, and 4 in the control group refused to continue. No more participants dropped out during the intervention and the follow-up assessments. As a result, 51 (98.08%) in the NAOM group, 48 (94.12%) in the COM group, and 48 (92.31%) in the control group completed all assessments and were included in the final analyses.

13b) For each group, losses and exclusions after randomisation, together with reasons

Does your paper address CONSORT subitem 13b? (NOTE: Preferably, this is shown in a CONSORT flow diagram) *

Copy and paste relevant sections from the manuscript (include quotes in quotation marks "like this" to indicate direct quotes from your manuscript), or elaborate on this item by providing additional information not in the ms, or briefly explain why the item is not applicable/relevant for your study

After randomization and allocation, 1 participant in the NAOM group, 3 in the COM group, and 4 in the control group refused to continue. No more participants dropped out during the intervention and the follow-up assessments. As a result, 51 (98.08%) in the NAOM group, 48 (94.12%) in the COM group, and 48 (92.31%) in the control group completed all assessments and were included in the final analyses.

13b-i) Attrition diagram

Strongly recommended: An attrition diagram (e.g., proportion of participants still logging in or using the intervention/comparator in each group plotted over time, similar to a survival curve) or other figures or tables demonstrating usage/dose/engagement.

	1	2	3	4	5	
subitem not at all important	○	○	○	○	○	essential

Does your paper address subitem 13b-i?

Copy and paste relevant sections from the manuscript or cite the figure number if applicable (include quotes in quotation marks "like this" to indicate direct quotes from your manuscript), or elaborate on this item by providing additional information not in the ms, or briefly explain why the item is not applicable/relevant for your study

您的回答

14a) Dates defining the periods of recruitment and follow-up

Does your paper address CONSORT subitem 14a? *

Copy and paste relevant sections from the manuscript (include quotes in quotation marks "like this" to indicate direct quotes from your manuscript), or elaborate on this item by providing additional information not in the ms, or briefly explain why the item is not applicable/relevant for your study

Participants were recruited from a faculty of nursing at a medical college in Beijing, China by using WeChat-based and offline publicity from March 1 to 16, 2024.

14a-i) Indicate if critical “secular events” fell into the study period

Indicate if critical “secular events” fell into the study period, e.g., significant changes in Internet resources available or “changes in computer hardware or Internet delivery resources”

	1	2	3	4	5	
subitem not at all important	○	○	○	○	○	essential

Does your paper address subitem 14a-i?

Copy and paste relevant sections from the manuscript (include quotes in quotation marks "like this" to indicate direct quotes from your manuscript), or elaborate on this item by providing additional information not in the ms, or briefly explain why the item is not applicable/relevant for your study

您的回答

14b) Why the trial ended or was stopped (early)

Does your paper address CONSORT subitem 14b? *

Copy and paste relevant sections from the manuscript (include quotes in quotation marks "like this" to indicate direct quotes from your manuscript), or elaborate on this item by providing additional information not in the ms, or briefly explain why the item is not applicable/relevant for your study

This trial did not end or be stopped early.

15) A table showing baseline demographic and clinical characteristics for each group

NPT: When applicable, a description of care providers (case volume, qualification, expertise, etc.) and centers (volume) in each group

Does your paper address CONSORT subitem 15? *

Copy and paste relevant sections from the manuscript (include quotes in quotation marks "like this" to indicate direct quotes from your manuscript), or elaborate on this item by providing additional information not in the ms, or briefly explain why the item is not applicable/relevant for your study

The demographic characteristics of the participants at baseline in different groups are summarized in Table 2. A total of 147 nursing students completed all assessments, with an average age of 20.44±3.06 years old. Among them, 79.59% were female, 91.84% were of Han ethnicity, 87.07% were undergraduate students, 40.14% held positions as student cadres, 51.70% were the only child in their family, 44.22% had an annual family income below 100,000 Yuan, 74.15% chose the nursing major on their own will, 21.09% had family members engaged in nursing, and 14.29% have ever been diagnosed with mental illness such as anxiety and depression. There were no significant cross-group differences in demographic characteristics in this study.

15-i) Report demographics associated with digital divide issues

In ehealth trials it is particularly important to report demographics associated with digital divide issues, such as age, education, gender, social-economic status, computer/Internet/ehealth literacy of the participants, if known.

	1	2	3	4	5	
subitem not at all important	○	○	○	○	○	essential

Does your paper address subitem 15-i? *

Copy and paste relevant sections from the manuscript (include quotes in quotation marks "like this" to indicate direct quotes from your manuscript), or elaborate on this item by providing additional information not in the ms, or briefly explain why the item is not applicable/relevant for your study

The demographic characteristics of the participants at baseline in different groups are summarized in Table 2. A total of 147 nursing students completed all assessments, with an average age of 20.44±3.06 years old. Among them, 79.59% were female, 91.84% were of Han ethnicity, 87.07% were undergraduate students, 40.14% held positions as student cadres, 51.70% were the only child in their family, 44.22% had an annual family income below 100,000 Yuan, 74.15% chose the nursing major on their own will, 21.09% had family members engaged in nursing, and 14.29% have ever been diagnosed with mental illness such as anxiety and depression. There were no significant cross-group differences in demographic characteristics in this study.

16) For each group, number of participants (denominator) included in each analysis and whether the analysis was by original assigned groups

16-i) Report multiple “denominators” and provide definitions

Report multiple “denominators” and provide definitions: Report N’s (and effect sizes) “across a range of study participation [and use] thresholds” [1], e.g., N exposed, N consented, N used more than x times, N used more than y weeks, N participants “used” the intervention/comparator at specific pre-defined time points of interest (in absolute and relative numbers per group). Always clearly define “use” of the intervention.

	1	2	3	4	5	
subitem not at all important	○	○	○	○	○	essential

Does your paper address subitem 16-i? *

Copy and paste relevant sections from the manuscript (include quotes in quotation marks "like this" to indicate direct quotes from your manuscript), or elaborate on this item by providing additional information not in the ms, or briefly explain why the item is not applicable/relevant for your study

Generalized estimation equation analysis
The results of the GEE analysis are summarized in Table 3. The parameter estimation of GEE analysis showed that interaction effects of depressive symptoms, anxiety symptoms, fatigue, and mindfulness were statistically significant, with P values < 0.001. Meanwhile, neither the interaction (time × group) nor a main effect of group difference was found in the quality of sleep, personality-Neuroticism, and resilience.
The scores of PHQ-9 and GAD-7 in all three groups decreased at the post-intervention assessment, and compared with before the intervention, the number of individuals with PHQ-9 and GAD-7 scoring 0-4 increased after the intervention, as shown in Figures 5 and 6. Except for resilience, all variables showed improvements after the six-week intervention, and even persisted until the end of the 3-month follow-up, differed significantly from the baseline assessment. Compared with the control group, the NAOM and COM group with the six-week online mindfulness program showed better effects in improving the level of mindfulness, and alleviating depression, anxiety, and fatigue symptoms after the intervention, and the difference was statistically significant(P<0.05). Moreover, significant differences in depression symptoms, anxiety symptoms, and level of mindfulness were also found between the NAOM group and the COM group at the post-intervention assessment (P<0.05). However, except for anxiety symptoms, there was no significant difference between the three groups at the 1-month and 3-month follow-up.

16-ii) Primary analysis should be intent-to-treat

Primary analysis should be intent-to-treat, secondary analyses could include comparing only “users”, with the appropriate caveats that this is no longer a randomized sample (see 18-i).

	1	2	3	4	5	
subitem not at all important	○	○	○	○	○	essential

Does your paper address subitem 16-ii?

Copy and paste relevant sections from the manuscript (include quotes in quotation marks "like this" to indicate direct quotes from your manuscript), or elaborate on this item by providing additional information not in the ms, or briefly explain why the item is not applicable/relevant for your study

您的回答

17a) For each primary and secondary outcome, results for each group, and the estimated effect size and its precision (such as 95% confidence interval)

Does your paper address CONSORT subitem 17a? *

Copy and paste relevant sections from the manuscript (include quotes in quotation marks "like this" to indicate direct quotes from your manuscript), or elaborate on this item by providing additional information not in the ms, or briefly explain why the item is not applicable/relevant for your study

Generalized estimation equation analysis
The results of the GEE analysis are summarized in Table 3. The parameter estimation of GEE analysis showed that interaction effects of depressive symptoms, anxiety symptoms, fatigue, and mindfulness were statistically significant, with P values < 0.001. Meanwhile, neither the interaction (time × group) nor a main effect of group difference was found in the quality of sleep, personality-Neuroticism, and resilience.
The scores of PHQ-9 and GAD-7 in all three groups decreased at the post-intervention assessment, and compared with before the intervention, the number of individuals with PHQ-9 and GAD-7 scoring 0-4 increased after the intervention, as shown in Figures 5 and 6. Except for resilience, all variables showed improvements after the six-week intervention, and even persisted until the end of the 3-month follow-up, differed significantly from the baseline assessment. Compared with the control group, the NAOM and COM group with the six-week online mindfulness program showed better effects in improving the level of mindfulness, and alleviating depression, anxiety, and fatigue symptoms after the intervention, and the difference was statistically significant(P<0.05). Moreover, significant differences in depression symptoms, anxiety symptoms, and level of mindfulness were also found between the NAOM group and the COM group at the post-intervention assessment (P<0.05). However, except for anxiety symptoms, there was no significant difference between the three groups at the 1-month and 3-month follow-up.

17a-i) Presentation of process outcomes such as metrics of use and intensity of use

In addition to primary/secondary (clinical) outcomes, the presentation of process outcomes such as metrics of use and intensity of use (dose, exposure) and their operational definitions is critical. This does not only refer to metrics of attrition (13-b) (often a binary variable), but also to more continuous exposure metrics such as “average session length”. These must be accompanied by a technical description how a metric like a “session” is defined (e.g., timeout after idle time) [1] (report under item 6a).

	1	2	3	4	5	
subitem not at all important	○	○	○	○	○	essential

Does your paper address subitem 17a-i?

Copy and paste relevant sections from the manuscript (include quotes in quotation marks "like this" to indicate direct quotes from your manuscript), or elaborate on this item by providing additional information not in the ms, or briefly explain why the item is not applicable/relevant for your study

您的回答

17b) For binary outcomes, presentation of both absolute and relative effect sizes is recommended

Does your paper address CONSORT subitem 17b? *

Copy and paste relevant sections from the manuscript (include quotes in quotation marks "like this" to indicate direct quotes from your manuscript), or elaborate on this item by providing additional information not in the ms, or briefly explain why the item is not applicable/relevant for your study

Binary outcomes was not applicable for this study.

18) Results of any other analyses performed, including subgroup analyses and adjusted analyses, distinguishing pre-specified from exploratory

Does your paper address CONSORT subitem 18? *

Copy and paste relevant sections from the manuscript (include quotes in quotation marks "like this" to indicate direct quotes from your manuscript), or elaborate on this item by providing additional information not in the ms, or briefly explain why the item is not applicable/relevant for your study

Generalized estimation equation analysis
The results of the GEE analysis are summarized in Table 3. The parameter estimation of GEE analysis showed that interaction effects of depressive symptoms, anxiety symptoms, fatigue, and mindfulness were statistically significant, with P values < 0.001. Meanwhile, neither the interaction (time × group) nor a main effect of group difference was found in the quality of sleep, personality-Neuroticism, and resilience.
The scores of PHQ-9 and GAD-7 in all three groups decreased at the post-intervention assessment, and compared with before the intervention, the number of individuals with PHQ-9 and GAD-7 scoring 0-4 increased after the intervention, as shown in Figures 5 and 6. Except for resilience, all variables showed improvements after the six-week intervention, and even persisted until the end of the 3-month follow-up, differed significantly from the baseline assessment. Compared with the control group, the NAOM and COM group with the six-week online mindfulness program showed better effects in improving the level of mindfulness, and alleviating depression, anxiety, and fatigue symptoms after the intervention, and the difference was statistically significant(P<0.05). Moreover, significant differences in depression symptoms, anxiety symptoms, and level of mindfulness were also found between the NAOM group and the COM group at the post-intervention assessment (P<0.05). However, except for anxiety symptoms, there was no significant difference between the three groups at the 1-month and 3-month follow-up.

18-i) Subgroup analysis of comparing only users

A subgroup analysis of comparing only users is not uncommon in ehealth trials, but if done, it must be stressed that this is a self-selected sample and no longer an unbiased sample from a randomized trial (see 16-iii).

	1	2	3	4	5	
subitem not at all important	○	○	○	○	○	essential

Does your paper address subitem 18-i?

Copy and paste relevant sections from the manuscript (include quotes in quotation marks "like this" to indicate direct quotes from your manuscript), or elaborate on this item by providing additional information not in the ms, or briefly explain why the item is not applicable/relevant for your study

您的回答

19) All important harms or unintended effects in each group

(for specific guidance see CONSORT for harms)

Does your paper address CONSORT subitem 19? *

Copy and paste relevant sections from the manuscript (include quotes in quotation marks "like this" to indicate direct quotes from your manuscript), or elaborate on this item by providing additional information not in the ms, or briefly explain why the item is not applicable/relevant for your study

There is no harm important or unintended effects in each group of this study.

19-i) Include privacy breaches, technical problems

Include privacy breaches, technical problems. This does not only include physical “harm” to participants, but also incidents such as perceived or real privacy breaches [1], technical problems, and other unexpected/unintended incidents. “Unintended effects” also includes unintended positive effects [2].

	1	2	3	4	5	
subitem not at all important	○	○	○	○	○	essential

Does your paper address subitem 19-i?

Copy and paste relevant sections from the manuscript (include quotes in quotation marks "like this" to indicate direct quotes from your manuscript), or elaborate on this item by providing additional information not in the ms, or briefly explain why the item is not applicable/relevant for your study

您的回答

19-ii) Include qualitative feedback from participants or observations from staff/researchers

Include qualitative feedback from participants or observations from staff/researchers, if available, on strengths and shortcomings of the application, especially if they point to unintended/unexpected effects or uses. This includes (if available) reasons for why people did or did not use the application as intended by the developers.

	1	2	3	4	5	
subitem not at all important	○	○	○	○	○	essential

Does your paper address subitem 19-ii?

Copy and paste relevant sections from the manuscript (include quotes in quotation marks "like this" to indicate direct quotes from your manuscript), or elaborate on this item by providing additional information not in the ms, or briefly explain why the item is not applicable/relevant for your study

您的回答

DISCUSSION

22) Interpretation consistent with results, balancing benefits and harms, and considering other relevant evidence

NPT: In addition, take into account the choice of the comparator, lack of or partial blinding, and unequal expertise of care providers or centers in each group

22-i) Restate study questions and summarize the answers suggested by the data, starting with primary outcomes and process outcomes (use)

Restate study questions and summarize the answers suggested by the data, starting with primary outcomes and process outcomes (use).

	1	2	3	4	5	
subitem not at all important	○	○	○	○	○	essential

Does your paper address subitem 22-i? *

Copy and paste relevant sections from the manuscript (include quotes in quotation marks "like this" to indicate direct quotes from your manuscript), or elaborate on this item by providing additional information not in the ms, or briefly explain why the item is not applicable/relevant for your study

This three-armed randomized controlled trial examined the effectiveness of a six-week online mindfulness program in enhancing the mental health of nursing students in China, and explored the ability of neurofeedback technology to enhance the effectiveness of the conventional online mindfulness intervention. Compared with the control group at the post-intervention, the 6-week online mindfulness intervention, both the neurofeedback-assisted and the conventional, resulted in significant decreases in depression, anxiety, and fatigue, as well as increases in mindfulness levels. Meanwhile, the results of this study indicated that the neurofeedback-assisted online mindfulness intervention was more effective than the conventional mindfulness in alleviating mental health problems in the short term after the intervention, and also had a positive effect on the long-term relief of anxiety symptoms. However, no other statistically significant differences between the NAOM group and the COM group were observed in the long term, indicating that the neurofeedback-assisted online mindfulness intervention has limited advantage in improving long-term mental health status in our study.

22-ii) Highlight unanswered new questions, suggest future research

Highlight unanswered new questions, suggest future research.

	1	2	3	4	5	
subitem not at all important	○	○	○	○	○	essential

Does your paper address subitem 22-ii?

Copy and paste relevant sections from the manuscript (include quotes in quotation marks "like this" to indicate direct quotes from your manuscript), or elaborate on this item by providing additional information not in the ms, or briefly explain why the item is not applicable/relevant for your study

您的回答

20) Trial limitations, addressing sources of potential bias, imprecision, and, if relevant, multiplicity of analyses

20-i) Typical limitations in ehealth trials

Typical limitations in ehealth trials: Participants in ehealth trials are rarely blinded. Ehealth trials often look at a multiplicity of outcomes, increasing risk for a Type I error. Discuss biases due to non-use of the intervention/usability issues, biases through informed consent procedures, unexpected events.

	1	2	3	4	5	
subitem not at all important	○	○	○	○	○	essential

Does your paper address subitem 20-i? *

Copy and paste relevant sections from the manuscript (include quotes in quotation marks "like this" to indicate direct quotes from your manuscript), or elaborate on this item by providing additional information not in the ms, or briefly explain why the item is not applicable/relevant for your study

This study has several limitations. First, the participants in the study were self-selected from a single medical college in Beijing, China, who were predominantly young, female, and tech-savvy college nursing students, limiting the generalizability of our results to the whole nursing students population in China, and to wider and more diverse populations. Second, the relatively small sample size might reduce the statistical power of the results; studies with larger samples covering more diverse populations are expected. Third, anxiety, depression, and other symptoms were assessed using self-reported measures, which may have been influenced by social desirability bias. Objective indicators such as electroencephalography and fMRI should be considered to comprehensively evaluate the intervention effects in the future. Fourth, the study was entirely quantitative, which should better be supplemented with qualitative research providing insights into the specific experiences of the participating nursing students regarding the interventions. Fifth, the participants were not asked to report if they have done any extra practice outside of the required lessons, which might be associated with intervention outcomes(69). Last, some possible mediators of the intervention effects were not investigated, such as mindfulness skills or rumination of the participants.

21) Generalisability (external validity, applicability) of the trial findings

NPT: External validity of the trial findings according to the intervention, comparators, patients, and care providers or centers involved in the trial

21-i) Generalizability to other populations

Generalizability to other populations: In particular, discuss generalizability to a general Internet population, outside of a RCT setting, and general patient population, including applicability of the study results for other organizations

	1	2	3	4	5	
subitem not at all important	○	○	○	○	○	essential

Does your paper address subitem 21-i?

Copy and paste relevant sections from the manuscript (include quotes in quotation marks "like this" to indicate direct quotes from your manuscript), or elaborate on this item by providing additional information not in the ms, or briefly explain why the item is not applicable/relevant for your study

您的回答

21-ii) Discuss if there were elements in the RCT that would be different in a routine application setting

Discuss if there were elements in the RCT that would be different in a routine application setting (e.g., prompts/reminders, more human involvement, training sessions or other co-interventions) and what impact the omission of these elements could have on use, adoption, or outcomes if the intervention is applied outside of a RCT setting.

	1	2	3	4	5	
subitem not at all important	○	○	○	○	○	essential

Does your paper address subitem 21-ii?

Copy and paste relevant sections from the manuscript (include quotes in quotation marks "like this" to indicate direct quotes from your manuscript), or elaborate on this item by providing additional information not in the ms, or briefly explain why the item is not applicable/relevant for your study

您的回答

OTHER INFORMATION

23) Registration number and name of trial registry

Does your paper address CONSORT subitem 23? *

Copy and paste relevant sections from the manuscript (include quotes in quotation marks "like this" to indicate direct quotes from your manuscript), or elaborate on this item by providing additional information not in the ms, or briefly explain why the item is not applicable/relevant for your study

The trial was prospectively registered on the Chinese Clinical Trial Registry (ID: ChiCTR2400080314) on January 25, 2024.

24) Where the full trial protocol can be accessed, if available

Does your paper address CONSORT subitem 24? *

Cite a Multimedia Appendix, other reference, or copy and paste relevant sections from the manuscript (include quotes in quotation marks "like this" to indicate direct quotes from your manuscript), or elaborate on this item by providing additional information not in the ms, or briefly explain why the item is not applicable/relevant for your study

The full trial protocol can be accessed on the website "the Chinese Clinical Trial Registry".

25) Sources of funding and other support (such as supply of drugs), role of funders

Does your paper address CONSORT subitem 25? *

Copy and paste relevant sections from the manuscript (include quotes in quotation marks "like this" to indicate direct quotes from your manuscript), or elaborate on this item by providing additional information not in the ms, or briefly explain why the item is not applicable/relevant for your study

FUNDING INFORMATION
This work was supported by Sanming Project of Medicine in Shenzhen (No. SZSM202211032).

X27) Conflicts of Interest (not a CONSORT item)

X27-i) State the relation of the study team towards the system being evaluated

In addition to the usual declaration of interests (financial or otherwise), also state the relation of the study team towards the system being evaluated, i.e., state if the authors/evaluators are distinct from or identical with the developers/sponsors of the intervention.

	1	2	3	4	5	
subitem not at all important	○	○	○	○	○	essential

Does your paper address subitem X27-i?

Copy and paste relevant sections from the manuscript (include quotes in quotation marks "like this" to indicate direct quotes from your manuscript), or elaborate on this item by providing additional information not in the ms, or briefly explain why the item is not applicable/relevant for your study

您的回答

About the CONSORT EHEALTH checklist

As a result of using this checklist, did you make changes in your manuscript? *

- ○ yes, major changes
- ◉ yes, minor changes
- ○ no

What were the most important changes you made as a result of using this checklist?

您的回答

How much time did you spend on going through the checklist INCLUDING making changes in your manuscript *

Spend about 60 minutes on going through the checklist.

As a result of using this checklist, do you think your manuscript has improved? *

- (●) yes
- ( ) no
- ( ) 其他:

Would you like to become involved in the CONSORT EHEALTH group?

This would involve for example becoming involved in participating in a workshop and writing an "Explanation and Elaboration" document

- ( ) yes
- ( ) no
- ( ) 其他:

Any other comments or questions on CONSORT EHEALTH

您的回答

STOP - Save this form as PDF before you click submit

To generate a record that you filled in this form, we recommend to generate a PDF of this page (on a Mac, simply select "print" and then select "print as PDF") before you submit it.

When you submit your (revised) paper to JMIR, please upload the PDF as supplementary file.

Don't worry if some text in the textboxes is cut off, as we still have the complete information in our database. Thank you!

Final step: Click submit !

Click submit so we have your answers in our database!

提交 清除表单内容

切勿通过 Google 表单提交密码。

此内容不是由 Google 所创建，Google 不对其作任何担保。 - 服务条款 - 隐私权政策

Does this form look suspicious? 报告

Google 表单
